# Comparing four heat-inducible promoters in stably transformed sugarcane regarding spatial and temporal control of transgene expression reveals candidates to drive stem-preferred transgene expression

**DOI:** 10.3389/fpls.2025.1709171

**Published:** 2025-12-03

**Authors:** Moni Qiande, Fredy Altpeter

**Affiliations:** 1 Agronomy Department, Plant Molecular and Cellular Biology Program, Genetics Institute, University of Florida, Institute of Food and Agricultural Sciences (IFAS), Gainesville, FL, United States; 2 DOE Center for Advanced Bioenergy and Bioproducts Innovation, Gainesville, FL, United States

**Keywords:** heat inducible promoter, sugarcane, vegetative tissue, uidA, stem, GUS activity, transgene expression, in silico analysis of heat shock elements

## Abstract

Small heat shock protein (sHSP) promoters contain *cis*-regulatory elements that facilitate transcription in response to heat stress, making them valuable tools for functional studies through controlled gene expression and the precise regulation of gene-editing tools or morphogenic regulators. To evaluate their utility, GUS reporter gene expression driven by four plant-sourced *HSP* promoters (p*GmHSP17.5*, p*HvHSP17*, p*ZmHSP17.7*, and p*ZmHSP26*) was compared across various tissues of stably transformed sugarcane before and after heat treatment. At 22°C, all promoters showed minimal activity in leaves and roots, although p*ZmHSP17.7* and p*HvHSP17* displayed moderate expression in stems. Following heat treatment, all promoters exhibited their highest activity in stems, followed by leaves and roots. In stem tissues, p*GmHSP17.5* displayed heat-induced *uidA* expression comparable to the constitutive p*ZmUbi* promoter. Notably, heat-induced reporter gene activity in stem middle sections of single-copy transgenic lines containing p*ZmHSP17.7*, p*HvHSP17*, or p*ZmHSP26* exceeded p*ZmUbi*-derived *uidA* activity by 9.7-fold, 3.8-fold, and 3.0-fold, respectively, with 346- to 3,672-fold induction compared to control conditions. Most promoters showed peak expression in the middle sections of the stem, while p*HvHSP17* was the most active in the stem apices. Histochemical analysis revealed that p*ZmHSP17.7* and p*HvHSP17* were active in both parenchyma cells and vascular bundles within sugarcane stems. Among leaf tissues, mature leaves exhibited greater expression than senescing or immature leaves, while root activity remained consistently minimal across all promoters. Temperature-course experiments identified distinct activation thresholds: 34°C–36°C for p*ZmHSP17.7*, 36°C for p*ZmHSP26*, 36°C–38°C for p*HvHSP17*, and 40°C–42°C for p*GmHSP17.5*. Drought stress also induced reporter gene transcription in stems under *HSP* promoters, although with lower fold induction than heat treatment. These findings provide valuable tools for gene function studies and biotechnology applications, including heat stress tolerance research, controlled transgene expression in metabolic engineering, precision gene editing, and developmental biology studies.

## Introduction

Climate change, driven by increased human activity since the Industrial Revolution, poses an imminent and far-reaching threat. Consequently, heat stress has emerged as an increasingly frequent challenge in crop production (IPCC, 2022). High temperatures can significantly alter plant growth and development at morphological, physiological, and molecular levels (Fahad et al., 2017; Li et al., 2023). In response to heat stress, plants produce heat shock proteins (HSPs), which are essential for maintaining cellular homeostasis by preserving protein conformation and preventing non-functional protein aggregation (Vierling, 1991; Wang et al., 2004).

HSPs are well-conserved between species and can be identified by their characteristic heat shock domain (Helm et al., 1993). All plant HSPs can also be categorized into one of five families based on their molecular weight (HSP100, HSP90, HSP70, HSP60, and small HSPs). Among these, the small heat shock protein (sHSP) family acts as molecular chaperones to stabilize protein folding and degrade misfolding proteins (Haslbeck et al., 2004, 1999; Lee et al., 1997). sHSPs are regulated under a variety of abiotic stresses, including heat (Howarth, 1991), drought (Grigorova et al., 2011), heavy metals (Györgyey et al., 1991), and osmotic stress (Almoguera et al., 1993; Coca et al., 1996). The overexpression of sHSPs has been shown to improve abiotic stress tolerance in crops (Feng et al., 2019; Sato and Yokoya, 2008).

Over the last few decades, focus has also been placed on the promoters of *HSP* genes. *HSP* promoters harbor multiple heat shock factor (HSF) binding sites. These are short, highly conserved motifs (5′-nGAAn-3′), also known as heat shock elements (HSEs). The inducible nature of such promoters provides various applications for plant biotechnology, including promoter strength evaluation (Freeman et al., 2011; Lyznik et al., 1995; Rerksiri et al., 2013), gene functional characterization (Wu et al., 2009), the activation of expression for gene-editing components (Barone et al., 2020; Nandy et al., 2019), and the controlled excision of transgenes by activating site-specific recombination systems (Akbudak and Srivastava, 2011; Sheva et al., 2020; Zhao et al., 2019; Khattri et al., 2011).

A well-characterized plant *HSP* promoter is the *Glycine max* (L.) *HSP17.5* promoter (p*GmHSP17.5*). p*GmHSP17.5* was identified using insertion/deletion mutagenesis (Czarnecka et al., 1989) and has been shown to drive stronger transgene expression after heat induction than with the constitutive 35S promoter (Ainley and Key, 1990). p*GmHSP17.5* has also been used to induce the CRISPR/Cas9 system for the generation of heritable mutations (Nandy et al., 2019) and to activate site-specific recombination in sugarcane (Zhao et al., 2019). Similar findings have also been demonstrated for the *Hordeum vulgare* (L.) *HSP17* promoter (p*HvHSP17*). p*HvHSP17* has two HSEs (Marmiroli et al., 1993; Raho et al., 1995) and was confirmed as heat-inducible in *Nicotiana tabacum* (Raho et al., 1996), *Zea mays* (Gullì et al., 2005), and *Triticum aestivum* L (Freeman et al., 2011). The *Zm*HSP26 protein has been identified (Nieto-Sotelo et al., 1990) and shown to be induced under heat stress in maize (Hu et al., 2015). The *ZmHSP26* promoter (p*ZmHSP26*) and *ZmHSP17.7* promoter (p*ZmHSP17.7*) have recently been documented to successfully activate a Cre-*lox* site-specific recombination system for the excision of selectable marker and morphogenic genes in *Z. mays* (Wang et al., 2020).

Sugarcane (*Saccharum* spp. hybrid) is the source of 40% of the global biofuel and 80% of the world’s table sugar production (Hoang et al., 2015). However, challenges associated with sugarcane’s polyploid genome make traditional breeding methods arduous, highlighting it as an ideal candidate for molecular improvement and research. While p*GmHSP*17.5 has previously been shown to induce FLPe/*Frt* for transgene excision in sugarcane (Zhao et al., 2019), the efficacy of p*GmHSP*17.5 has not been compared with that of other *HSP* promoters. The current study examined four different *HSP* promoters (p*GmHSP17.5*, p*HvHSP17*, p*ZmHSP17.7*, and p*ZmHSP26*) in stably transformed sugarcane using the *uidA* gene encoding β-glucuronidase (GUS) as a reporter gene. GUS is a commonly used reporter system in plant biotechnology studies. Its expression does not negatively impact plant growth and development and supports both histochemical and quantitative analysis (Vain, 2007). We evaluated the strengths of the four *HSP* promoters at the GUS activity level using histochemical GUS assays and quantitative MUG assays (Jefferson et al., 1987) in leaf, stem, and root tissues with and without heat induction, and we investigated their activating temperatures at the transcriptional level with qRT-PCR in sugarcane leaves. We also examined the efficacies of *HSP* promoters under drought stress in sugarcane stems. This study produced new quantitative knowledge on the temporal and spatial expression of *HSP* promoters in sugarcane under heat and drought, thus expanding the promoter toolbox for crop biotechnology.

## Methods

### Vector construction and gene transformation

**Figure 1:**
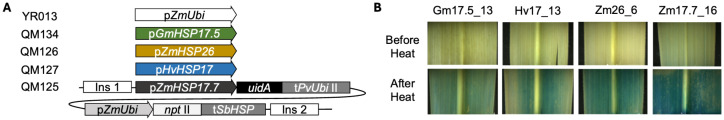
**(A)** Transgene cassettes used for transformation: p*ZmHSP17.7*, promoter of *Zea mays* HEAT SHOCK PROTEIN *17.7* gene; p*ZmHSP26*, promoter of *Z. mays* HEAT SHOCK PROTEIN *26* gene; p*HvHSP17*, promoter of *Hordeum vulgare* HEAT SHOCK PROTEIN *17* gene; p*GmHSP17.5*, promoter of *Glycine max* HEAT SHOCK PROTEIN *17.5* gene; p*ZmUbi*, promoter of *Z. mays UBI*QUITIN gene; *uidA*, β-glucuronidase gene; t*PvUbi*II, *Panicum virgatum* ubiquitin terminator; p*ZmUbi*, *Z. mays* ubiquitin promoter; *npt* II, neomycin phosphotransferase II; t*SbHSP*, *Sorghum bicolor* HEAT SHOCK PROTEIN terminator; Ins, insulator. **(B)** Representative leaf GUS staining results of different *HSP* lines before and after the heat treatment.

Four vectors, each containing a *uidA* expression cassette and a reporter gene cassette, were constructed using the Golden Gate cloning method (Engler et al., 2014). The coding sequence of *uidA* was codon-optimized for sugarcane using custom gene synthesis (GenScript, NJ, USA). For the *uidA* expression cassettes, p*GmHSP17.5*, p*HvHSP17*, p*ZmHSP17.7*, p*ZmHSP26*, and p*ZmUbi* (abbreviated as *Ubi*) were used to drive *uidA* in vectors QM134, QM127, QM125, QM126, and YR013, respectively (**Figure 1A**). In the reporter gene cassettes, *neomycin phosphotransferase* II (*npt* II) was used as a selectable marker driven by p*ZmUbi*. Two nuclear matrix attachment regions (MARs) from *N. tabacum* were used as insulators to flank the linked expression cassettes (Allen et al., 1996; Xue et al., 2005). The vector backbone was removed *via* overnight restriction digestion using *I-SceI* (New England Biolabs, MA, USA). Transgene fragments were electrophoresed and purified using a GeneJET Gel Purification Kit (Thermo Fisher Scientific, MA, USA), coated onto gold microparticles, and delivered to callus cultures of sugarcane cultivar CP88–1762 using biolistics as described by Sandhu and Altpeter (2008).

### Plant material and conditions

The sugarcane tops of cultivar CP88–1762 were collected from field-grown sugarcane at the grand growth stage from the UF-IFAS Plant Research and Education Unit located near Citra, Florida. Callus induction was performed using immature leaf whorls for indirect embryogenesis as described by Kim et al. (2012). All culture media were prepared according to Kim et al. (2012). Once roots were established, regenerated plantlets (V0 generation; vegetative 0 generation) were transferred to soil and cultivated in a greenhouse setting. All plants under greenhouse conditions were grown in 22-cm-diameter pots, receiving 600 mL of irrigation per day via a drip irrigation system, with natural light and temperatures being controlled with air conditioning to 16°C to 20°C at night and 21°C to 25°C during the day. V0 plants (1m in height) were sampled for genomic DNA extraction.

### Copy number assay

Genomic DNA was extracted using the cetyltrimethylammonium bromide (CTAB) method (Murray and Thompson, 1980). Ten microliters of TaqMan^®^ Gene Expression Master Mix, 1 μL of the customized TaqMan^®^ probe (Applied Biosystems^®^, Thermo Fisher Scientific Inc., MA, USA), 7 μL of DNase/RNase-free water, and 20 ng of genomic DNA were used (20 μL total volume) to detect *uidA* copy number under the following conditions on a CFX connect system (Bio-Rad, CA, USA): denaturation for 10min at 95°C and 40 cycles of 15 s at 95°C and 60 s at 60°C. Sugarcane rust resistance gene (*Bru*1) was used for the normalization of the *uidA* gene copy number. Data were analyzed using the Applied Biosystems^®^ CopyCaller^®^ v2.1 software (Applied Biosystems, Thermo Fisher Scientific, MA, USA) following the manufacturer’s guidance. Primers used for copy number assay are listed in **Supplementary Table S1**.

### Heat and drought stress treatments and sample collection

Heat treatments were conducted in a TPRB growth room (BioChambers Incorporated, MB, Canada) located at the UF/IFAS Growth Chamber Facility. Preliminary heat treatments consisted of heating 1-m-tall V0 plants for 2h (8:00 am to 10:00 am) at 40°C. Before and after heat treatment, two samples were collected from the middle of the first dewlap leaves of V0 plants. The mature node segments of V0 plants were used to generate the V1 generation under controlled greenhouse conditions as stated above.

Once exceeding 1.5m in height, V1 plants were sampled for fluorometric GUS assays (MUG assay) from the second dewlap leaves, stems (top of stem, middle of stem, and base of stem) of different tillers, and roots. A 4-day heat treatment was then completed, which contained four heat cycles, each 4 h long (from 8:00 am to 12:00 pm every day) at 40°C, 40% relative humidity, and 1,125 μmol m^−2^ s^−1^ Photosynthetic Photon Flux Density (PPFD) light intensity. During non-heat-treated hours, conditions were set to 22°C and 75% humidity. Day length was set for 15 h from 5:00 am to 8:00 pm with 1,125 μmol m^−2^ s^−1^ PPFD light intensity. After treatment, samples were collected from the middle sections of immature leaves, first dewlap leaves, third dewlap leaves, fifth dewlap leaves, stems (top of stem, middle of stem, and base of stem), and roots. Wild-type (WT) and *HSP* lines (V1 generation) were then vegetatively propagated from node cuttings to generate V2 progenies and biological replicates. V2 progenies were grown under controlled greenhouse conditions as stated above.

A subset of V2 plants was treated with drought stress by shutting off irrigation. Stem samples (top of stem, middle of stem, and base of stem) were collected when the relative water content (RWC %) in the potting soil reached 50% (mild drought) or 20% (severe drought). RWC % was measured using the FieldScout TDR 350 Soil Moisture Meter (Spectrum Technologies, Bridgend, UK).

A heat cycle (from 8:00 am to 10:00 am) was conducted to measure the minimal and optimal activation temperatures of *HSP* promoters. Another subset of V2 plants was split into six groups and received a 2-h heat treatment at 34°C, 36°C, 38°C, 40°C, 42°C, and 44°C, and the middle sections of the first dewlap leaves were sampled for RNA extraction.

Each treatment/genotype was sampled as three biological replicates, except for the V0 samples.

### Histochemical GUS assay and counterstaining

A histochemical GUS assay was conducted to visualize GUS localization based on Jefferson et al. (1987). Tissues were immersed in GUS staining solution (**Supplementary Table S2**) and incubated for 48h at 37°C. The GUS-treated tissues were then incubated in 70% ethanol at room temperature to remove chlorophyll. Counterstaining was conducted following the procedures stated in Kim et al. (2002). Photos were captured using a ZEISS Axiocam 305 color microscope (ZEISS, Oberkochen, Germany).

### Fluorometric GUS assay

Fluorometric GUS assays were conducted to quantify GUS activity based on Jefferson et al. (1987). A 4-methylumbelliferone (4-MU) standard curve was performed as follows: emissions of 1,000, 500, 150, 50, 20, 10, 5, and 1 nM 4-MU standards diluted in 0.2 M Na_2_CO_3_ were measured at 365 nm for excitation and 455 nm for emission using the BioTek Synergy H1 hybrid reader (Agilent Technologies, CA, USA). Protein quantification was conducted using Quick Start™ Bradford 1× Dye reagent (Bio-Rad, CA, USA) following the manufacturer’s instructions. A bovine serum albumin (BSA) standard curve was created with 2, 1.5, 1, 0.75, 0.5, 0.25, and 0.125 mg/mL BSA standards diluted in GUS extraction buffer (GEB) (**Supplementary Table S2**) and measured for absorbance at 595 nm. For MUG assays, mixtures of 20 μL of crude extract and 180 μL of AMB (**Supplementary Table S2**) were incubated at 37°C in the dark. After 30min of incubation, 100 μL of 0.2 M Na_2_CO_3_ was added to stop enzyme activity, and the mixtures were measured at 365 nm for excitation and 455 nm for emission. GUS activity was calculated in pmol 4-MU/(min·mg).

### Quantitative RT-PCR analysis

RNA was extracted using TRIzol™ Reagent (Thermo Fisher Scientific, MA, USA), following the manufacturer’s protocol. One microgram of extracted RNA was used to obtain cDNA using a High-Capacity cDNA Reverse Transcription Kit (Applied Biosystems, MA, USA). Quantitative PCR (qPCR) was conducted with SsoAdvanced Universal SYBR green supermix (Bio-Rad, CA, USA) according to the manufacturer’s guidance using a CFX connect system (Bio-Rad, CA, USA). qPCR conditions were as follows: denaturation for 3min at 95°C, 40 cycles of 10 s at 95°C and 30 s at 60°C, and melting for 10 s at 95°C followed by 0.5°C increment temperature increase every 5 s from 65°C to 95°C. *GLYCERALDEHYDE 3-PHOSPHATE DEHYDROGENASE* (*GAPDH*) was used to normalize *uidA* gene expression. *uidA* relative expression = 2^{Ct (GAPDH)−Ct (transgene)}^. Primers used for qPCR are listed in **Supplementary Table S1**.

### *In silico* analysis of heat shock elements in the different *HSP* promoters

The sequences of the four *HSP* promoters were analyzed for HSE configurations using PlantPan3.0 (Chow et al., 2019), PlantCARE (Lescot et al., 2002), and PlantTFDB (PlantTFDB v5.0) (Tian et al., 2020). Putative HSEs were also called using motif pattern searches encompassing variants with two or three tandemly alternating repeats of nGAAn, allowing one to two nucleotide substitutions in the core GAA motif and/or one to two bp insertions between the repeats. Python codes used for HSE sequence calling were deposited at https://github.com/qiandemoni/HSE_sequence_finder. Motifs with two alternating repeats of nGAAn, no mismatches, and no insertions were considered minimal HSE. Detected imperfect HSEs were ranked according to the type and number of substitutions in the core motif, number of bp insertions between motifs, and distance from the transcription start site (TSS).

### Statistical analyses

Statistical analysis was completed using ANOVA in GraphPad Prism (version 10.0.1). The least significant difference (LSD) method was used for the comparisons of means. Paired Student’s t-test was used to analyze the effect of mild/severe drought stresses within the same lines.

## Results

### Generation of low-copy-number GUS-expressing transgenic sugarcane lines

Linearized vectors containing *HSP* promoters driving *uidA* expression cassettes and *npt* II selectable marker cassettes flanked by insulators were delivered into sugarcane calli (**Figure 1A**). Thirty-one independent *HSP* promoter V0 transgenic lines were generated with seven to nine lines per construct (**Supplementary Table S3**). Twelve of the lines (two to six lines for each of the constructs) were identified to contain a single-copy *uidA* insertion, with the remaining 20 displaying between two and five copies (**Supplementary Table S3**). GUS assay was conducted in all lines before and after a preliminary 2-h heat treatment at 40°C (**Supplementary Figure S1**). For further analysis, five p*GmHSP17.5* lines, five p*HvHSP17* lines, five p*ZmHSP26* lines, and four p*ZmHSP17.7* lines were selected based on the before- and after-heat GUS staining. The selected *HSP* lines displayed growth and development similar to WT plants (**Supplementary Figure S2**). Histochemical GUS staining before and after heat treatment in the representative *HSP* lines also confirmed that all four promoters were sufficient to induce GUS expression in sugarcane leaves (**Figure 1B**). Two single-copy *uidA* lines driven by a *ZmUbi* promoter were used as constitutive controls.

### Heat shock element analysis

Promoter analysis revealed substantial variation in HSE organization among the four *HSP* promoters. HSE motifs can be highly variable without compromising their function during the heat stress response. Neither PlantCARE nor PlantPAN3.0 provides a dedicated category for HSF-binding sites. PlantTFDB search included HSE motifs. However, it generated markedly fewer hits than those identified through a customized HSE motif search (**Supplementary Tables S4**, **S5**). Performing a customized HSE motif search revealed no canonical HSEs (nGAAnnTTCnnGAAn or nTTCnnGAAnnTTCn) for any of the four evaluated *HSP* promoters, but different groups of imperfect HSEs (**Supplementary Figure S3**). Allowing one to two nucleotide substitutions in the core GAA motif and/or one to two bp insertions between the three tandemly alternating repeats of the nGAAn motif will likely attenuate but not abolish the heat response. These criteria resulted in 23, 17, 10, and 10 imperfect HSEs for *pGmHSP17.5*, *pZmHSP17.7*, *pHvHSP17.7*, and *pZmHSP26*, respectively, in the customized motif search (**Supplementary Table S5**). The minimal partially functional HSE is represented by two alternating pentamers (nGAAnnTTCn or nTTCnnGAAn), for which two, one, two, and one perfect hits were identified in *pGmHSP17.5*, *pZmHSP17.7*, *pHvHSP17.7*, and *pZmHSP26*, respectively (**Supplementary Table S5**). Allowing one nucleotide substitution in the core GAA motif and/or one to two bp insertions between the two tandemly alternating repeats of the nGAAn motif resulted in 41, 69, 26, and 20 imperfect minimal HSEs in *pGmHSP17.5*, *pZmHSP17.7*, *pHvHSP17.7*, and *pZmHSP26*, respectively (**Supplementary Table S5**). Twelve, 13, two, and one of these minimal and imperfect HSE motifs were located within 100 bp upstream of the TSS of *pGmHSP17.5*, *pZmHSP17.7*, *pHvHSP17.7*, and *pZmHSP26*, respectively (**Supplementary Table S5**). Combining the different criteria, our custom motif search resulted in a total of 66, 87, 38, 31 non-canonical HSEs in *pGmHSP17.5*, *pZmHSP17.7*, *pHvHSP17.7*, and *pZmHSP26*, respectively, and were ranked according to their potential functionality in heat response (**Supplementary Figure S3**).

### GUS activities in different leaf positions following heat induction

**Figure 2:**
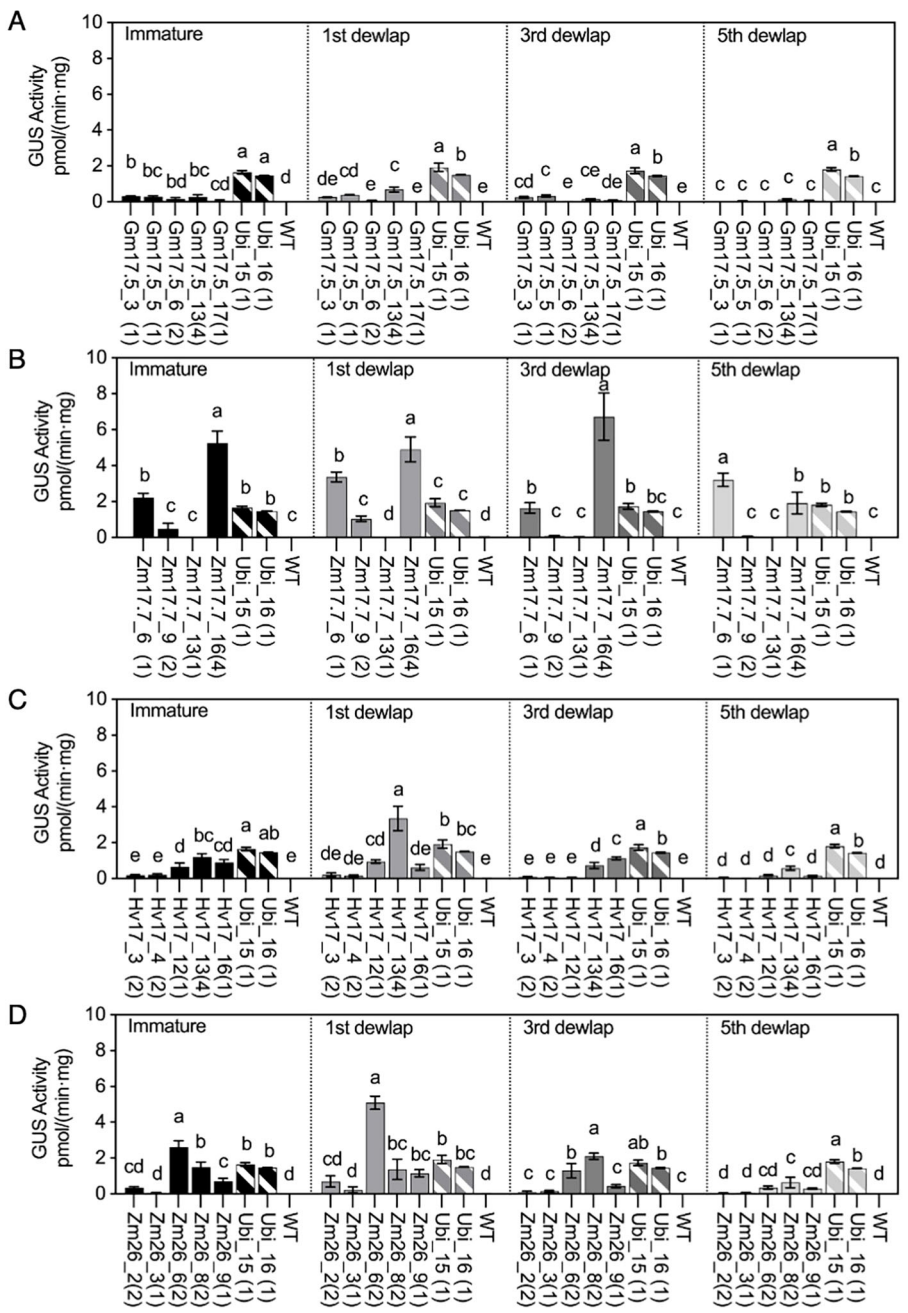
GUS activities in *HSP* lines and wild type (WT) after heat treatment compared with those in *Ubi* promoter lines under normal greenhouse conditions in different leaf positions (immature leaf, first dewlap leaf, third dewlap leaf, and fifth dewlap leaf). **(A)** p*GmHSP17.5* lines, **(B)** p*ZmHSP17.7* lines, **(C)** p*HvHSP17* lines, and **(D)** p*ZmHSP26* lines. *uidA* copy numbers are shown in parentheses following the line IDs. Solid bars indicate the *HSP* lines; shadowed bars indicate the *Ubi* promoter lines. Error bars indicate standard error. One-way ANOVA was conducted among different lines at same leaf positions, and different letters indicate significant difference at *p* < 0.05 according to Fisher’s least significant difference (LSD) comparison.

Trace amounts of GUS activity were detected in the second dewlap leaves of V1 WT plants, ranging from 0.01 to 0.03 and 0.00 to 0.02 pmol/(min·mg) before and after the 4-day heat treatment, respectively (**Figure 2A**, **Supplementary Table S6**). In the *HSP* lines, before-heat GUS activity was observed in the range of 0.00 to 0.10 pmol/(min·mg) (**Supplementary Table S6**), and this became elevated in all leaf tissues post-heat (**Figure 2A**). After heat treatment, the p*GmHSP17.5*, p*ZmHSP17.7*, p*ZmHSP26*, and p*HvHSP17* lines exhibited 0.41, 3.87, 1.15, and 2.41 pmol/(min·mg) GUS activity levels per *uidA* copy, respectively, on average of all leaf positions (**Figures 2A–D**). The fold changes in GUS activity in leaves before and after heat treatment ranged from 4.2- to 4,665.9-fold for p*GmHSP17.5*, 3.0- to 172.8-fold for p*ZmHSP17.7*, 1.6- to 1,137.2-fold for p*ZmHSP26*, and 1.2- to 56.4-fold for p*HvHSP17* lines (**Figures 2A–D**, **Supplementary Table S6**). In lines Gm17.5_13, Zm17.7_6, Zm26_6, and Hv17_13, the highest GUS activity was detected in the first dewlap leaf (**Figures 2A–D**). Conversely, in lines Zm17.7_16, Zm26_8, and Hv17_16, peak GUS activity was observed in the third dewlap leaves (**Figures 2B–D**). Lower GUS activity was observed in immature and fifth dewlap leaves for all the *HSP* lines (**Figures 2A–D**). In contrast, *Ubi* promoter lines showed consistent GUS activity levels across various leaf positions (**Figure 2A**). In lines Zm17.7_6, Zm26_6, and Hv17_13, GUS activity in the first dewlap leaves exceeded that of constitutive Ubi_15 and Ubi_16 controls (**Figures 2B–D**). However, for all leaf positions, p*GmHSP17.5* lines showed lower GUS activity compared to *Ubi* promoter lines (**Figure 2A**).

**Figure 3:**
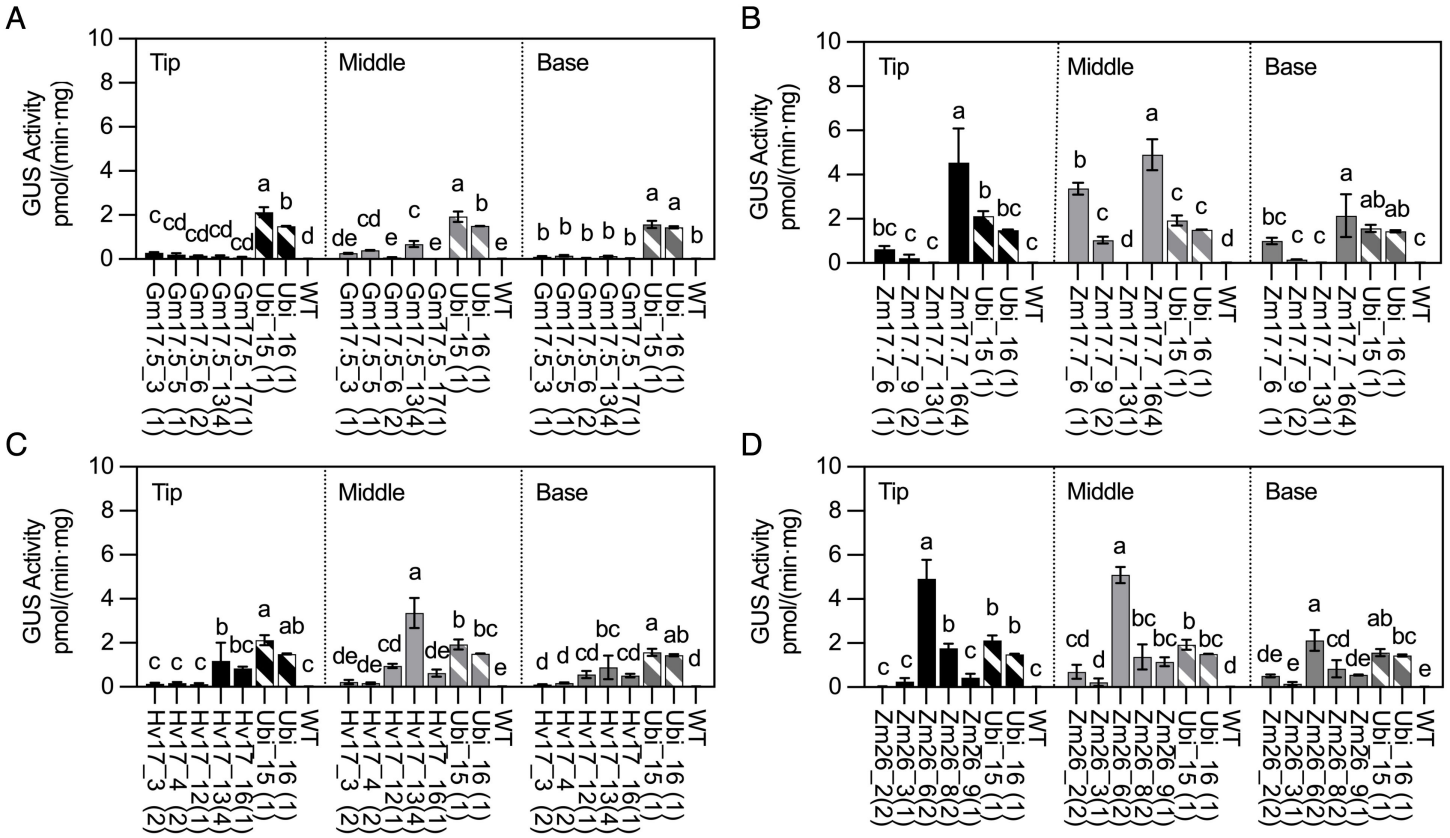
GUS activities in *HSP* lines and wild type (WT) after heat treatment compared with those in *Ubi* promoter lines under normal greenhouse conditions in first dewlap leaves (tip leaf section, middle leaf section, and base leaf section). **(A)** p*GmHSP17.5* lines, **(B)** p*ZmHSP17.7* lines, **(C)** p*HvHSP17* lines, and **(D)** p*ZmHSP26* lines. *uidA* copy numbers are shown in parentheses following the line IDs. Solid bars indicate the *HSP* lines; shadowed bars indicate the *Ubi* promoter lines. Error bars indicate standard error. One-way ANOVA was conducted among different lines at same leaf sections, and different letters indicate significant difference at *p* < 0.05 according to Fisher’s least significant difference (LSD) comparison.

The gradient of GUS activity within the first dewlap leaves (tip, middle, and base) was also investigated (**Figures 3A–D**). Most lines, including all the p*ZmHSP17.7* lines, displayed a trend of leaf middle sections having the highest GUS activity (**Figures 3A–D**). However, for lines Gm17.5_3, Hv17_16, and Zm26_8, the highest GUS activity was observed at the tip of the leaf (**Figures 3A, C, D**).

### GUS activity in different stem positions before and after heat induction

**Figure 4:**
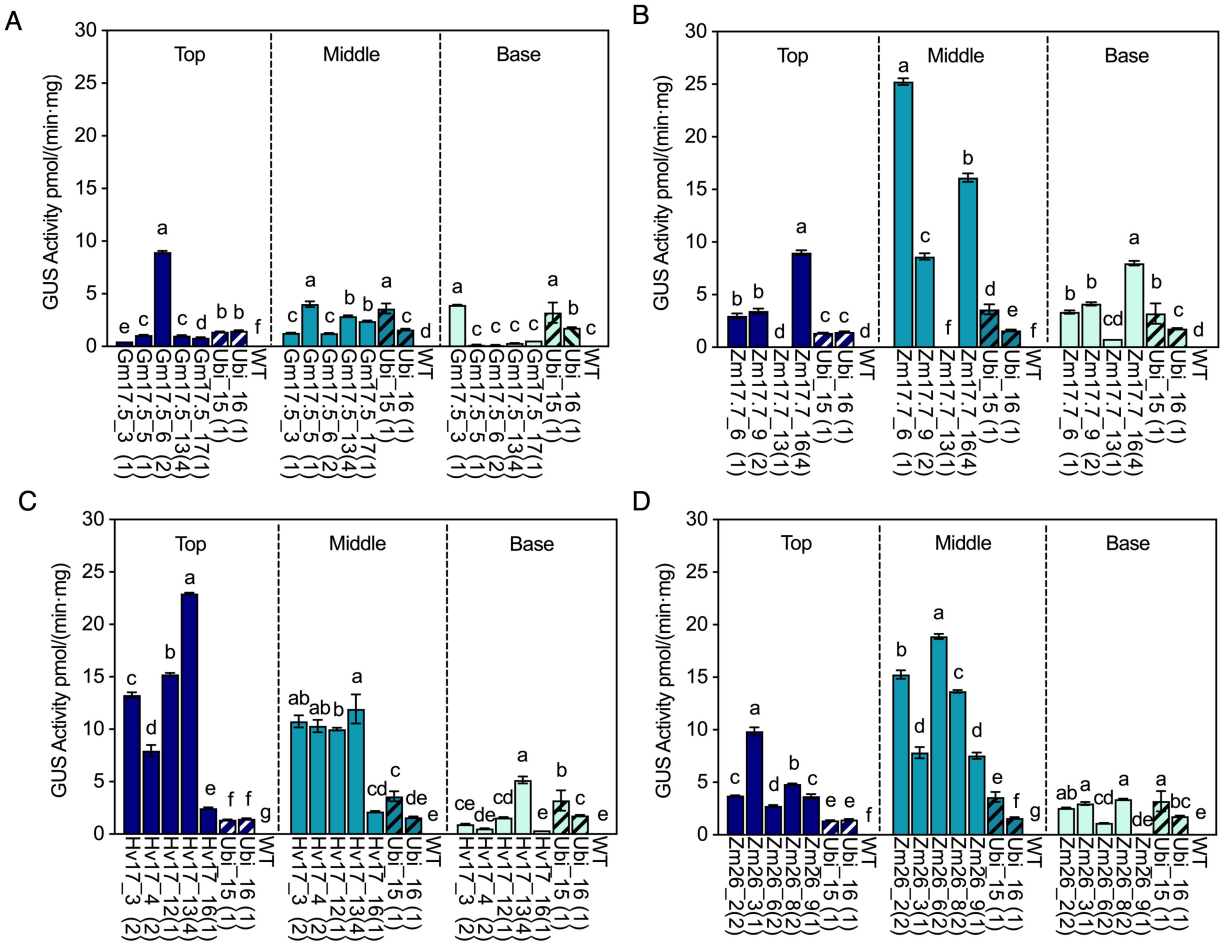
GUS activities in *HSP* lines and wild type (WT) after heat treatment compared with those in *Ubi* promoter lines under normal greenhouse conditions in different stem sections (top of stem, middle of stem, and base of stem). **(A)** p*GmHSP17.5* lines, **(B)** p*ZmHSP17.7* lines, **(C)** p*HvHSP17* lines, and **(D)** p*ZmHSP26* lines. *UidA* copy numbers are shown in parentheses following the line IDs. The solid bars indicate the *HSP* lines; the shadowed bars indicate the *Ubi* lines. Error bars indicate standard error. One-way ANOVA was conducted among different lines at same stem sections, and different letters indicate significant difference at *p* < 0.05 according to Fisher’s least significant difference (LSD) comparison.

WT, *HSP* lines (V1 generation), and *Ubi* promoter lines were investigated for their GUS activity in the top, middle, and base positions of the stem. Negligible GUS activity was observed in any position of the stems in WT, p*GmHSP17.5*, and p*ZmHSP26* lines prior to heat treatment (**Supplementary Figure S4**). However, some of the p*ZmHSP17.7* and p*HvHSP17* lines displayed GUS activity before heat treatment. In Hv17_13 and Zm17.7_6, the highest GUS activity levels before heat treatment were found in the middle and base of the stem, reaching 49.6% and 23.8% of that of the constitutive control line Ubi_16, respectively (**Supplementary Figure S4**). In WT, trace amounts of GUS activity, reaching up to 0.01 pmol/(min·mg), were detected in all stem positions after heat treatment (**Figure 4**). All *HSP* lines showed elevated GUS activity in the top, middle, and base of stem sections after heat (**Figure 4**). The before- and after-heat GUS activity fold changes were 32.3- to 3671.7-fold, 1.2- to 345.6-fold, 20.4- to 1567.6-fold, and 1.2- to 407.5-fold in all stem sections of all the p*GmHSP17.5*, p*ZmHSP17.7*, p*ZmHSP26*, and p*HvHSP17* lines, respectively (**Figure 4**). All the p*ZmHSP17.7* and p*ZmHSP26* lines showed the highest GUS activity in the middle sections of the stems (**Figures 4B, D**), while p*HvHSP17* lines displayed the highest activity at the top of the stems (**Figure 4C**). Gm17.5_6 showed peak GUS activity at the top of the stems, while GUS activity peaked in the middle of the stems for the rest of the p*GmHSP17.5* lines (**Figure 4A**). Overall, the absolute GUS activity after heat treatment in the middle stem was up to 103.0-, 8.3-, 34.6-, and 63.2-fold greater than that in the first dewlap leaves in p*GmHSP17.5*, p*ZmHSP17.7*, p*ZmHSP26*, and p*HvHSP17* lines, respectively (**Figures 2**, **4**). Compared to the average of two *Ubi* promoter lines, after-heat GUS activity in the middle stem was up to 1.5-, 9.7-, 7.2-, and 4.6-fold greater in p*GmHSP17.5*, p*ZmHSP17.7*, p*ZmHSP26*, and p*HvHSP17* lines, respectively (**Figure 4**).

### Roots do not display elevated GUS expression following heat treatment

In WT roots, GUS activity was 0.07 and 0.06 pmol/(min·mg) before and after heat treatment, respectively (**Supplementary Figure S5**). GUS activity before heat treatment in the roots of transgenic *HSP* lines was slightly higher than that in the leaves and stems, ranging from 0.02 to 0.11 pmol/(min·mg) (**Supplementary Figure S5**, **Supplementary Table S6**). However, the after-heat root GUS activity ranged similarly to that before heat treatment between 0.05 and 0.12 pmol/(min·mg), which was significantly lower than that of the two Ubi lines, which ranged from 1.74 to 3.41 pmol/(min·mg) (**Supplementary Figure S5**). While an elevated trend was observed in root GUS activity for some lines following heat induction, the change was not significant, indicating a lack of significant reporter gene activation in roots with the applied heat treatment (**Supplementary Figure S5**).

### Drought induces *HSP* promoters in sugarcane stem

**Figure 5:**
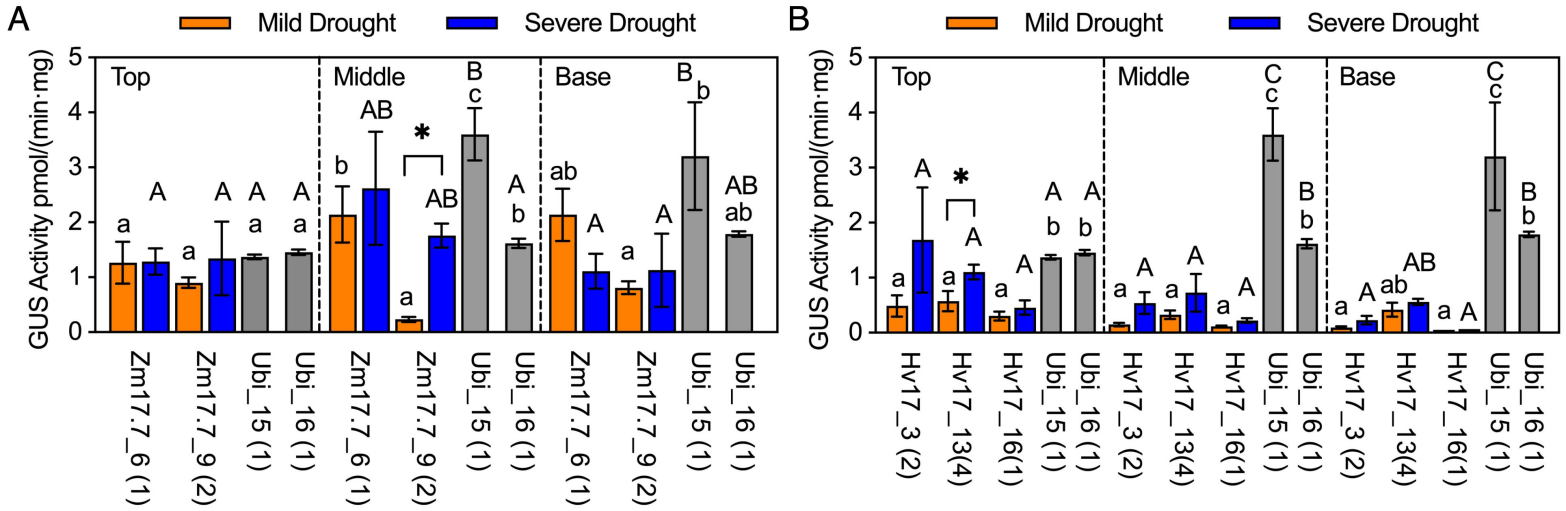
GUS activities in *HSP* lines after drought treatment compared with those in *Ubi* promoter lines under normal greenhouse conditions in different stem sections (top of stem, middle of stem, and base of stem). **(A)** p*ZmHSP17.7* lines and **(B)** p*HvHSP17* lines. Gray bars indicate *Ubi* lines. *uidA* copy numbers are shown in parentheses following the line IDs. Error bars indicate standard error. One-way ANOVA was conducted among different lines (*HSP* lines and *Ubi* lines) at same stem sections, and different upper/lowercase letters indicate significant difference at *p* < 0.05 in mild/severe drought treatment according to Fisher’s least significant difference (LSD) comparison. Paired Student’s t-test was conducted to compare the values after mild or severe drought treatment within the same line, and the significance was indicated by * (*p*<0.05).

After *HSP* lines from V2 generation reached a height of 1.5m, selected lines from p*ZmHSP17.7*, p*HvHSP17*, and p*ZmHSP26* were subjected to drought, and samples were collected following mild and severe drought stress. Compared to before drought treatment, the increases in GUS activity after the severe drought stress were 2.3- to 54.7-fold, 0.2- to 27.4-fold, and 6.6- to 31.1-fold in p*ZmHSP17.7*, p*ZmHSP26*, and p*HvHSP17* lines, respectively (**Figure 5**, **Supplementary Figure S4**). Notable increases of 3.5- and 7.5-fold were also observed between mild and severe drought stress in the top stem sections of Hv17_3 and the middle stem sections of Zm17.7_9 (**Figure 5**), yet for the rest of the lines, GUS activity measurements showed no significant differences between mild and severe drought stress (**Figure 5**, **Supplementary Figure S6**). The result of histochemical staining and counterstaining revealed that p*ZmHSP17.7* and p*HvHSP17* were active in both vascular bundles and parenchyma cells in sugarcane stems, while p*ZmHSP26* was mostly active in vascular bundles (**Supplementary Figure S7**, **Supplementary Figure S8**).

### Activation temperatures for gene expression driven by different *HSP* promoters vary

**Figure 6:**
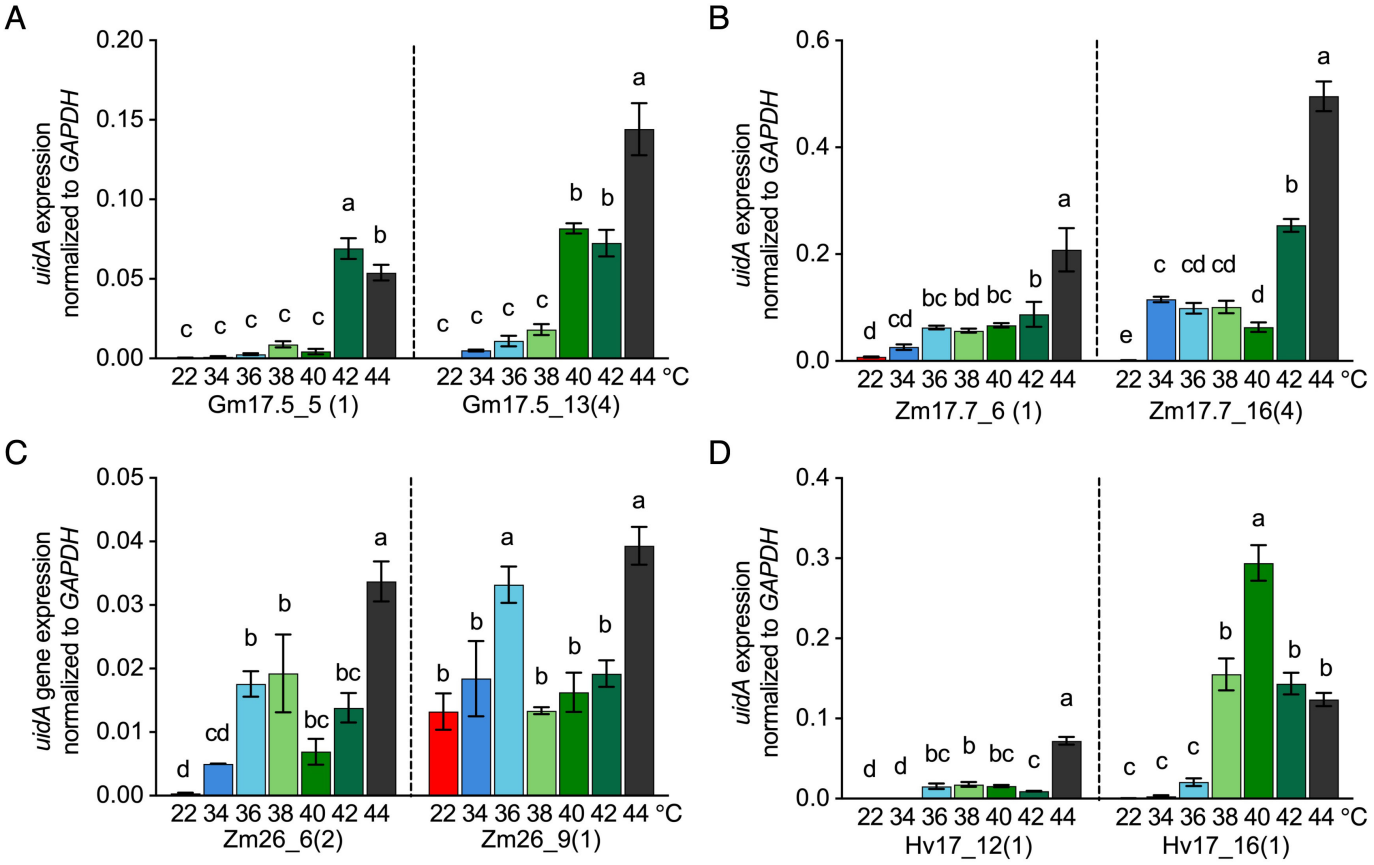
*uidA* expression normalized to housekeeping gene *GAPDH* after 2-h treatment at different temperatures (22°C, 34°C, 36°C, 38°C, 40°C, 42°C, and 44°C) in **(A)** p*GmHSP17.5* lines, **(B)** p*ZmHSP17.7* lines, **(C)** p*ZmHSP26* lines, and **(D)** p*HvHSP17* lines. *uidA* copy numbers are shown in parentheses following the line IDs. Values were derived from three biological replicates (n = 3). Error bars indicate standard error. One-way ANOVA was conducted, and different letters indicate significance at *p*<0.05 within the same line according to Fisher’s least significant difference (LSD) comparison.

The minimal and optimal induction temperatures for each *HSP* promoter were investigated using qRT-PCR at the transcriptional level in sugarcane leaf tissue. *HSP* lines from the V2 generation were heat-treated at temperatures ranging from 34°C to 44°C (2°C interval) for 2h in comparison to the 22°C control temperature. Significant transcription activation of *uidA* compared to the control temperature of 22°C was observed for the single-copy *uidA* lines of pZ*mHSP17.7* at 34°C to 36°C, p*ZmHSP26* at 36°C, for p*HvHSP17* at 36°C to 38°C, and for p*GmHSP17.5* at 42°C (**Figures 6A–D**). The highest *uidA* expression in these lines was detected between 40°C and 44°C (**Figures 6A–D**). The lines with four copies of p*GmHSP17.5* or p*ZmHSP17.7* displayed significant *uidA* expression induction at lower temperatures and approximately two- to threefold higher maximum expression at 44°C than the corresponding single-copy lines (**Figures 6A, B**).

## Discussion

Inducible promoters provide remarkable utility when sustained transgene expression compromises plant development or agronomic performance. Well-characterized *HSP* promoters enable heat- and/or drought-inducible transgene expression for diverse applications, including metabolic engineering, site-specific recombination, gene editing with reduced off-target effects, and engineering enhanced heat and drought stress tolerance.

This study quantitatively and histochemically examined the spatial and temporal expression patterns of a GUS reporter gene under the transcriptional control of four *HSP* promoters in stably transformed sugarcane, comparing their performance to a constitutive maize ubiquitin promoter. The results demonstrate that *HSP* promoters induce stronger expression in mature leaf tissues compared to immature or senescing tissues.

Following heat treatment, leaves from multiple p*ZmHSP17.7*, p*ZmHSP26*, and p*HvHSP17* transgenic lines displayed higher GUS activity than Ubi promoter lines, while all pGmHSP17.5 lines exhibited relatively weak GUS activity. These findings suggest that *HSP* promoters derived from monocotyledonous species (maize and barley) outperform those from dicotyledonous species (soybean) in sugarcane leaf tissues following heat induction.

Typically, leaves are expected to be more efficient at producing recombinant proteins than stems due to their distinct biochemical compositions. Sugarcane stems, for example, are rich in carbohydrates such as soluble sugars and lignocellulose and produce fewer proteins than sugarcane leaves (Palaniswamy et al., 2016). However, the results of this study indicate that differential regulation occurs in the leaves and stems of sugarcane following heat induction of *HSP* promoters.

Remarkably, sugarcane stems, which constitute approximately 70% of the plant’s biomass (Palaniswamy et al., 2016), displayed higher GUS activity than leaves after heat treatment. Similarly, a report in rice indicated that HSP-driven expression in the panicle was up to more than twofold higher than in the leaf (Rerksiri et al., 2013). Our findings have notable commercial value, as *HSP* promoters that enhance recombinant protein accumulation in sugarcane stems can be utilized for producing value-added proteins or expressing enzymes that catalyze hyper-accumulation of commercially important products, such as energy-dense lipids (Cao et al., 2023; Padilla et al., 2020; Parajuli et al., 2020). This approach also aligns with practical considerations, as existing sugar processing infrastructure for stem harvesting and processing can be readily adapted for these applications.

Prior to stress treatment, moderate levels of GUS activity were observed in the p*ZmHSP17.7* and p*HvHSP17* lines in sugarcane stems, whereas GUS activity driven by p*ZmHSP26* and p*GmHSP17.5* in stems was negligible. Most earlier reports have described that reporter gene activity or transcripts under the control of different *HSP* promoters are non-significant or undetectable prior to heat activation (Faralli et al., 2015; Freeman et al., 2011; Kuo et al., 2000; Pegoraro et al., 2011). However, several reports have described that some of the *HSP* promoters initiate transcripts or reporter gene activity under non-stress conditions, including during seed maturation (Prändl et al., 1995; Wehmeyer and Vierling, 2000) in the leaves of monocots (Harrington et al., 2020) and dicots (Bang et al., 2015; Khurana et al., 2013), the stigmas of monocots (Harrington et al., 2020), and the stems of dicots (Khurana et al., 2013).

Following drought treatment, the promoters p*ZmHSP17.7*, p*ZmHSP26*, and p*HvHSP17* also activated GUS activity in sugarcane stems. GUS expression was localized in both the storage parenchyma cells and vascular bundles of p*ZmHSP17.7* and p*HvHSP17* lines, and in the vascular bundles of p*ZmHSP26* lines. In comparison, in stable transgenic tobacco, GUS expression driven by p*HvHSP17* was strictly restricted to the xylem tissues of stems and petioles after heat induction (Raho et al., 1996). Although GUS staining of some heat-shocked tissues, including leaves, glumes, and palea/lemma, in transgenic wheat lines was more intense in vascular bundles, GUS expression was not confined to vascular bundles in these tissues, with expression also observed in the internodes and nodes of stems. However, the relative expression difference between stem and leaf tissues was not quantified (Freeman et al., 2011; Raho et al., 1995). Similar to our study, Coca et al. reported higher reporter gene activity in stems than in leaves following heat activation when driven by the *HaHSP17.7* promoter (Coca et al., 1996). However, in contrast to our observations, they found expression mainly in xylem and phloem rather than in parenchyma, and they did not observe upregulation by drought stress.

Fold inductions driven by *HSP* promoters following drought were substantially lower compared to those observed after heat treatment. This aligns with previous reports, which found that the expression of sHSPs, including HSP26, was more enhanced by heat than drought in maize seedlings (Hu et al., 2010). The HSP70 promoter from *Oryza sativa* also exhibited lower inducibility under drought compared to heat in rice (Rerksiri et al., 2013). In contrast, the GHSP26 gene was 100-fold more abundant in drought-stressed leaves, while only twofold more abundant in dehydrated stem and root compared to control tissues (Maqbool et al., 2007).

In a previous study, p*HvHSP17* induced GUS activity in the roots of heat-treated wheat seedlings (Freeman et al., 2011). However, in mature sugarcane HSP lines, heat exposure did not significantly elevate GUS activity in roots. The lack of reporter gene activation in sugarcane roots may be due to decreased temperature exposure, as the thermal insulation of soil slows heat penetration (Katan, 1981). Freeman et al. (2011) used a temperature-controlled hydroponic system, which overcomes this limitation, but the hydroponic approach is less relevant for field performance than the approach we chose.

We also investigated the minimal and optimal activating temperatures of the four *HSP* promoters in sugarcane leaves. The results indicated that p*ZmHSP17.7* and p*ZmHSP26* were highly induced at approximately 44°C, requiring only a short heat pulse. This aligns with previous findings in maize (Wang et al., 2020), where site-specific transgene excision with Cre-lox under the transcriptional control of p*ZmHSP17.7* or p*ZmHSP26* was the most successful at 42–45°C. Similarly, p*GmHSP17.5* was highly induced at 42–44°C. In contrast, p*HvHSP17* was induced at a lower temperature (38°C–40°C), consistent with results found in wheat seedlings (Freeman et al., 2011). These findings could be invaluable for tailoring gene expression systems to specific thermal profiles, using different *HSP* promoters for genes that require activation under varied temperature conditions.

Comparing our *in vivo* results with *in silico* analyses of HSEs confirmed that the promoter performance of heat shock proteins cannot be reliably predicted from sequence-based HSE analysis alone. The abundance of canonical HSEs detected *in silico* within *HSP* promoters did not correspond to their heat-induced activation levels observed *in vivo*. For instance, *pGmHSP17.5*, which contained more high-confidence, non-canonical HSE motifs than the other promoters, did not exhibit the highest GUS induction following heat treatment. Despite the considerable degeneracy and variability among HSE motifs, many still support HSF binding with differing affinities, making it difficult for motif-based algorithms to distinguish functional from non-functional sites. Current *in silico* motif searches identify sequences in isolation, without considering chromatin context, cooperative HSF binding, epigenetic states, interactions with other regulatory elements, or nucleosome positioning—all of which strongly influence promoter activity (Huang et al., 2023; Abdulraheem et al., 2024; Fragkostefanakis et al., 2025) and may account for the comparatively high inducibility of *pZmHSP17.7*.

Heat-inducible promoters, such as *HSP* promoters, can open new avenues for breeding stress-resistant crop species. One notable example is that heat-inducible expression of miRNA398 enhanced the heat tolerance of *Arabidopsis* plants (Guan et al., 2013). *HSP* promoters are also particularly useful for complex metabolic engineering, where multiple transgenes need to be co-expressed to exert a synergistic impact on the desired phenotype. The inducible expression of specific transgenes allows the elucidation of their relative contribution to the phenotype in the context of constitutively co-expressed contributing factors by comparing phenotypes before and after induction. The availability of multiple well-characterized *HSP* promoters with similar induction profiles, such as in this study, also facilitates transgene stacking by decreasing risks associated with the repeated use of the same regulatory element, including unintended recombination and gene silencing (Matzke et al., 1994).

## Conclusion

In this study, four different plant heat shock protein promoters were characterized in the vegetative tissues of stably transformed sugarcane to evaluate their efficacy and spatial expression profiles when directing the expression of a *uidA* reporter gene. Notably, p*ZmHSP17.7*, p*HvHSP17*, and p*ZmHSP26* drove several-fold higher heat-induced reporter gene activity in stems compared to the constitutive p*ZmUbi* promoter. The knowledge presented here will facilitate breeding for heat stress resilience and the development of traits requiring inducible transgene expression for complex metabolic engineering applications.

## Data Availability

The data presented in the study are deposited in the Zenodo repository under the following record locator: https://zenodo.org/records/17486962.

## Supplementary material

Supplementary Figure 1 GUS staining of 1st dewlap leaves of all the transgenic *HSP* lines (V0) before and after 2h heat treatment at 40°C.

Supplementary Figure 2 The selected *HSP* lines (V1) next to the wildtype sugarcane (WT) before any treatments.

Supplementary Figure 3 *In silico* analysis of *HSP* promoter sequence revealing heat shock factor (HSF) binding sites containing two and three pentamers. Conservation levels of imperfect HSE motifs are “1”, “2A”, “2B”, “3”, “4A”, and “4B” as described below. 1: very likely to retain function (mildly reduced affinity/induction): no substitutions at conserved GAA or TTC positions of 2 pentamers (nGAAnnTTCn or nTTCnnGAAn) and may have a third pentamer with substitutions. No insertions between pentamers. 2A: likely to retain partial function (reduced affinity/induction): substitutions within a GAA where the substituted base is an A (i.e., one of the A’s but not the G) or single substitutions within a TTC where the substituted base is T (position 2) or C (position 3) with no insertions between pentamers. 2B: likely to retain partial function (reduced affinity/induction): no substitutions at conserved GAA or TTC positions of 2 pentamers (nGAAnnTTCn or nTTCnnGAAn) and may have a third pentamer with substitutions. 1–2 bp Insertions between pentamers. 3: less likely to retain partial function (more reduced affinity/induction): substitutions within a GAA where the substituted base is an A (i.e., one of the A’s but not the G) or single substitutions within a TTC where the substituted base is T (position 2) or C (position 3). 1–2 bp insertions between pentamers. 4A: least likely to retain partial function (most reduced affinity/induction): substitutions of the conserved G within a GAA (G→X at positions 2) or substitutions of the T in the TTC core (T→X at positions 2), without insertions between pentamers. 4B: least likely to retain partial function (most reduced affinity/induction): substitutions of the conserved G within a GAA (G→X at positions 2) or substitutions of the T in the TTC core (T→X at positions 2), with 1–2 bp insertions between pentamers. Data refers to **Supplementary Table S5**.

Supplementary Figure 4 GUS activities in *HSP* lines and WT before heat treatment compared with that in *Ubi* promoter lines under normal greenhouse conditions in different stem sections (top of stem, middle of stem, and base of stem). *uidA* copy numbers were shown in parentheses following line IDs. The solid bars indicate the *HSP* lines; the shadowed bars indicate the *Ubi* lines. Error bars indicate standard error. One-way ANOVA was conducted among different lines at same stem sections, and different letters indicate significant difference at *p < *0.05 according to Fisher’s least significant difference (LSD) comparison.

Supplementary Figure 5 GUS activities in *HSP* lines and WT before and after heat treatment compared with that in *Ubi* promoter lines under normal greenhouse conditions in roots. *uidA* copy numbers were shown in parentheses following line IDs. The solid bars indicate the *HSP* lines; the shadowed bars indicate the *Ubi* lines. Error bars indicate standard error. One-way ANOVA was conducted among different lines at same stem sections, and different letters indicate significant difference at *p < *0.05 according to Fisher’s least significant difference (LSD) comparison.

Supplementary Figure 6 GUS activities in pZm*HSP26* lines after drought treatment compared with that in *Ubi* lines under normal greenhouse conditions (gray bars) in different stem sections (top of stem, middle of stem, and base of stem). *uidA* copy numbers were shown in parentheses following line IDs. Error bars indicate standard error. One-way ANOVA was conducted among different lines (*HSP* lines and *Ubi* lines) at same stem sections, and different upper/lowercase letters indicate significant difference at *p < *0.05 in mild/severe drought treatment according to Fisher’s least significant difference (LSD) comparison. Paired Student’s T test was conducted to compare the values after mild or severe drought treatment within the same line, and the significance was indicated by * (*p <* 0.05).

Supplementary Figure 7 GUS staining in different stem sections of the selected *HSP* lines after mild and severe drought stress. *uidA* copy numbers are shown in parentheses following line IDs.

Supplementary Figure 8 GUS counter-staining in different stem sections of the selected *HSP* lines after mild (50% RWC) and severe drought (20%RWC) stress. Pictures were taken at 5-10X magnitude and 40X magnitude. *uidA* copy numbers are shown in parentheses following line IDs.
