# Supplementary material for: Comparing four heat-inducible promoters in stably transformed sugarcane regarding spatial and temporal control of transgene expression reveals candidates to drive stem-preferred transgene expression
Source: Front Plant Sci. 2025 Dec 3;16:1709171. doi: 10.3389/fpls.2025.1709171 (PMC12708596; doi:10.3389/fpls.2025.1709171)
Supplement: Supplementary Figure 1 — GUS staining of 1st dewlap leaves of all the transgenic HSP lines (V0) before and after 2h heat treatment at 40°C. [file Presentation1.pptx]

## Slide 1
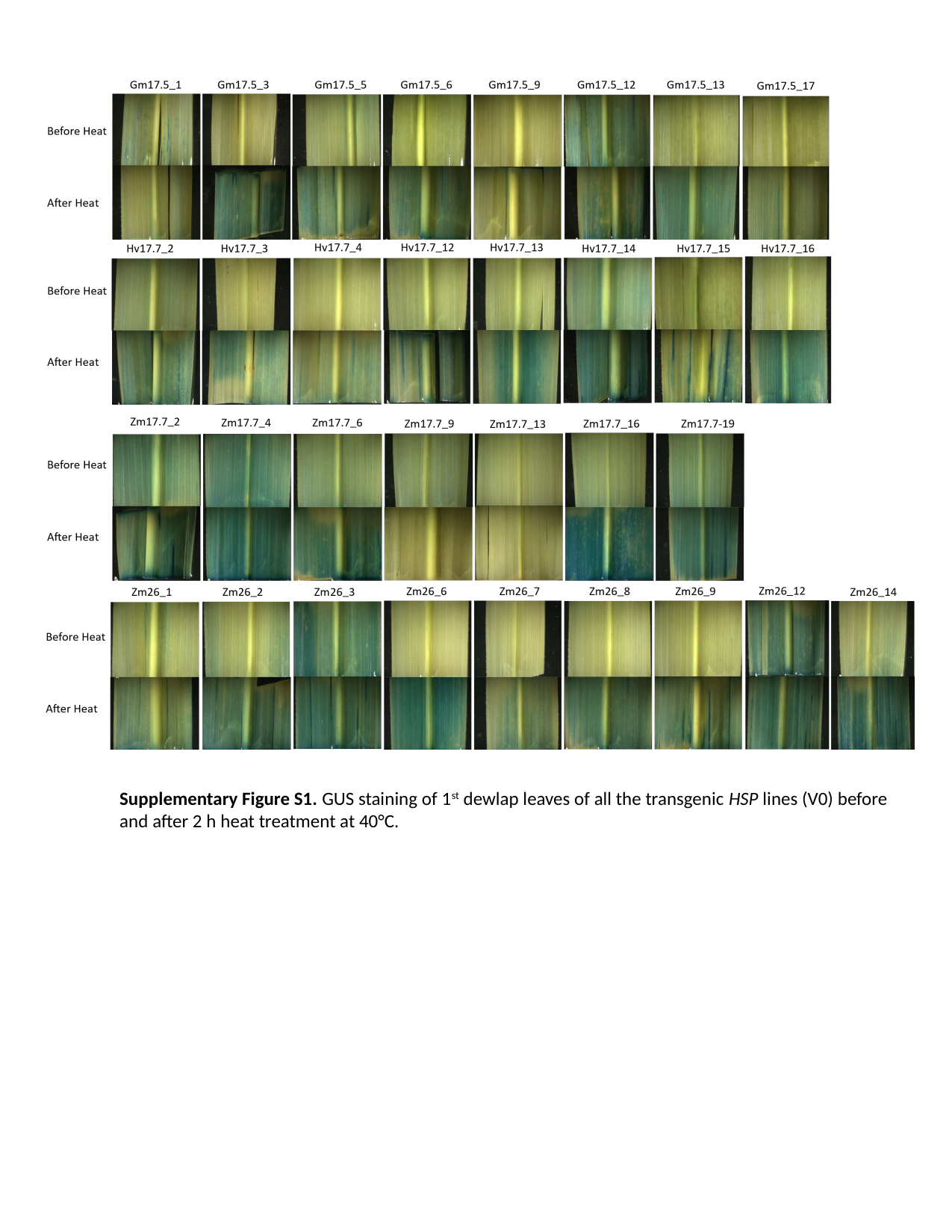

Supplementary Figure S1. GUS staining of 1st dewlap leaves of all the transgenic HSP lines (V0) before and after 2 h heat treatment at 40°C.

## Slide 2
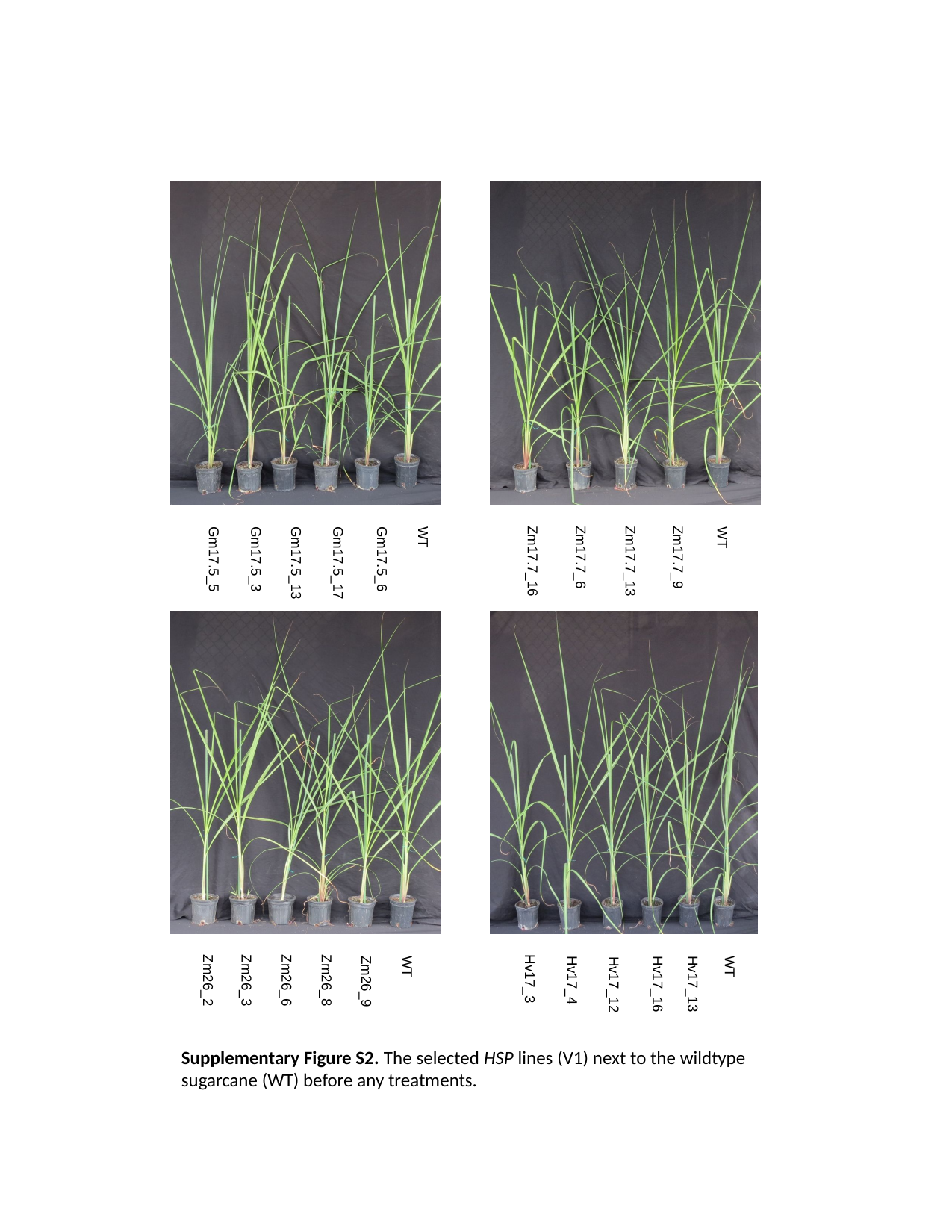

Gm17.5_6
WT
Gm17.5_13
Gm17.5_17
Gm17.5_5
Gm17.5_3
Zm17.7_13
Zm17.7_9
Zm17.7_6
Zm17.7_16
WT
Zm26_8
Zm26_6
Zm26_3
Zm26_2
Zm26_9
WT
Hv17_3
Hv17_13
WT
Hv17_4
Hv17_16
Hv17_12
Supplementary Figure S2. The selected HSP lines (V1) next to the wildtype sugarcane (WT) before any treatments.

## Slide 3
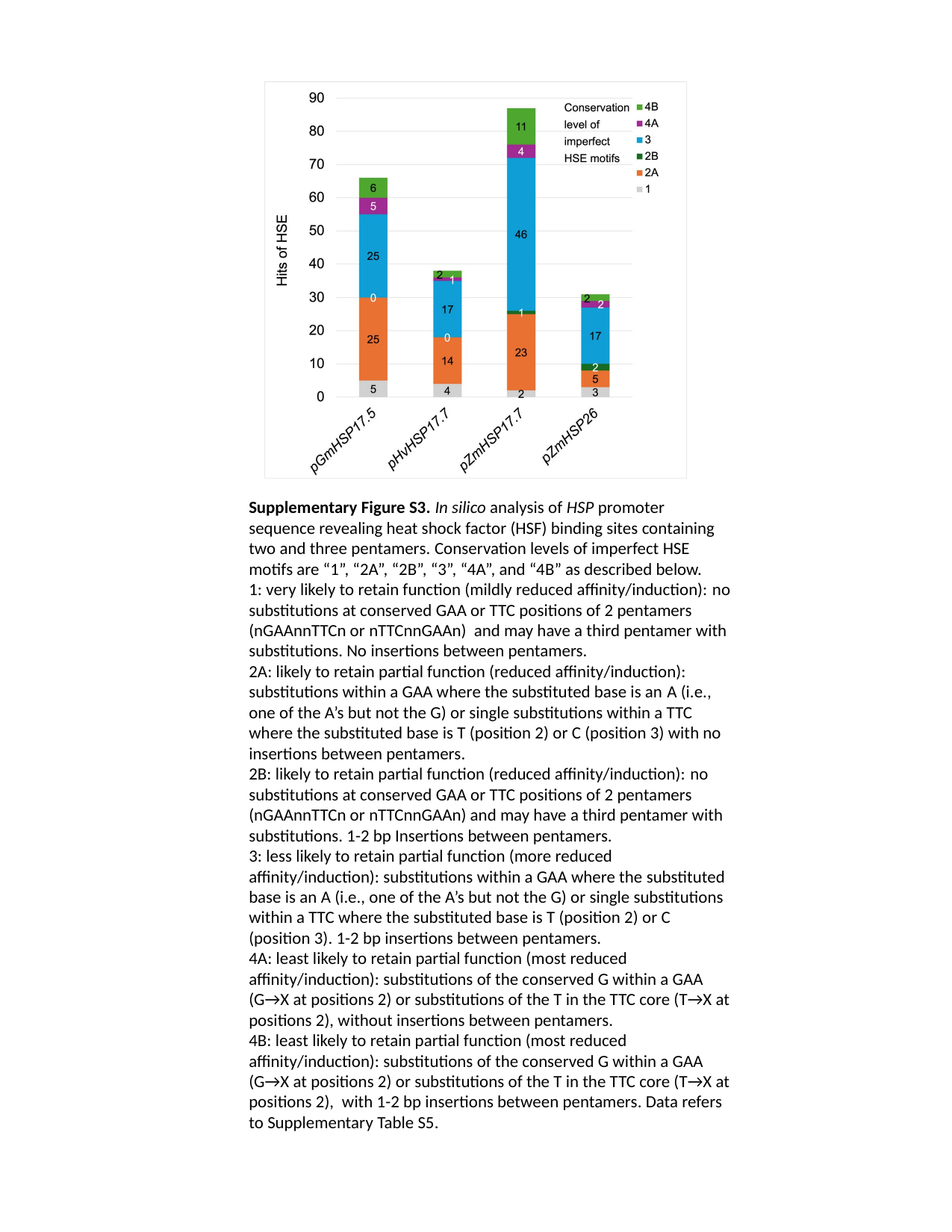

Supplementary Figure S3. In silico analysis of HSP promoter sequence revealing heat shock factor (HSF) binding sites containing two and three pentamers. Conservation levels of imperfect HSE motifs are “1”, “2A”, “2B”, “3”, “4A”, and “4B” as described below.
1: very likely to retain function (mildly reduced affinity/induction): no substitutions at conserved GAA or TTC positions of 2 pentamers (nGAAnnTTCn or nTTCnnGAAn) and may have a third pentamer with substitutions. No insertions between pentamers.
2A: likely to retain partial function (reduced affinity/induction): substitutions within a GAA where the substituted base is an A (i.e., one of the A’s but not the G) or single substitutions within a TTC where the substituted base is T (position 2) or C (position 3) with no insertions between pentamers.
2B: likely to retain partial function (reduced affinity/induction): no substitutions at conserved GAA or TTC positions of 2 pentamers (nGAAnnTTCn or nTTCnnGAAn) and may have a third pentamer with substitutions. 1-2 bp Insertions between pentamers.
3: less likely to retain partial function (more reduced affinity/induction): substitutions within a GAA where the substituted base is an A (i.e., one of the A’s but not the G) or single substitutions within a TTC where the substituted base is T (position 2) or C (position 3). 1-2 bp insertions between pentamers.
4A: least likely to retain partial function (most reduced affinity/induction): substitutions of the conserved G within a GAA (G→X at positions 2) or substitutions of the T in the TTC core (T→X at positions 2), without insertions between pentamers.
4B: least likely to retain partial function (most reduced affinity/induction): substitutions of the conserved G within a GAA (G→X at positions 2) or substitutions of the T in the TTC core (T→X at positions 2),  with 1-2 bp insertions between pentamers. Data refers to Supplementary Table S5.

## Slide 4
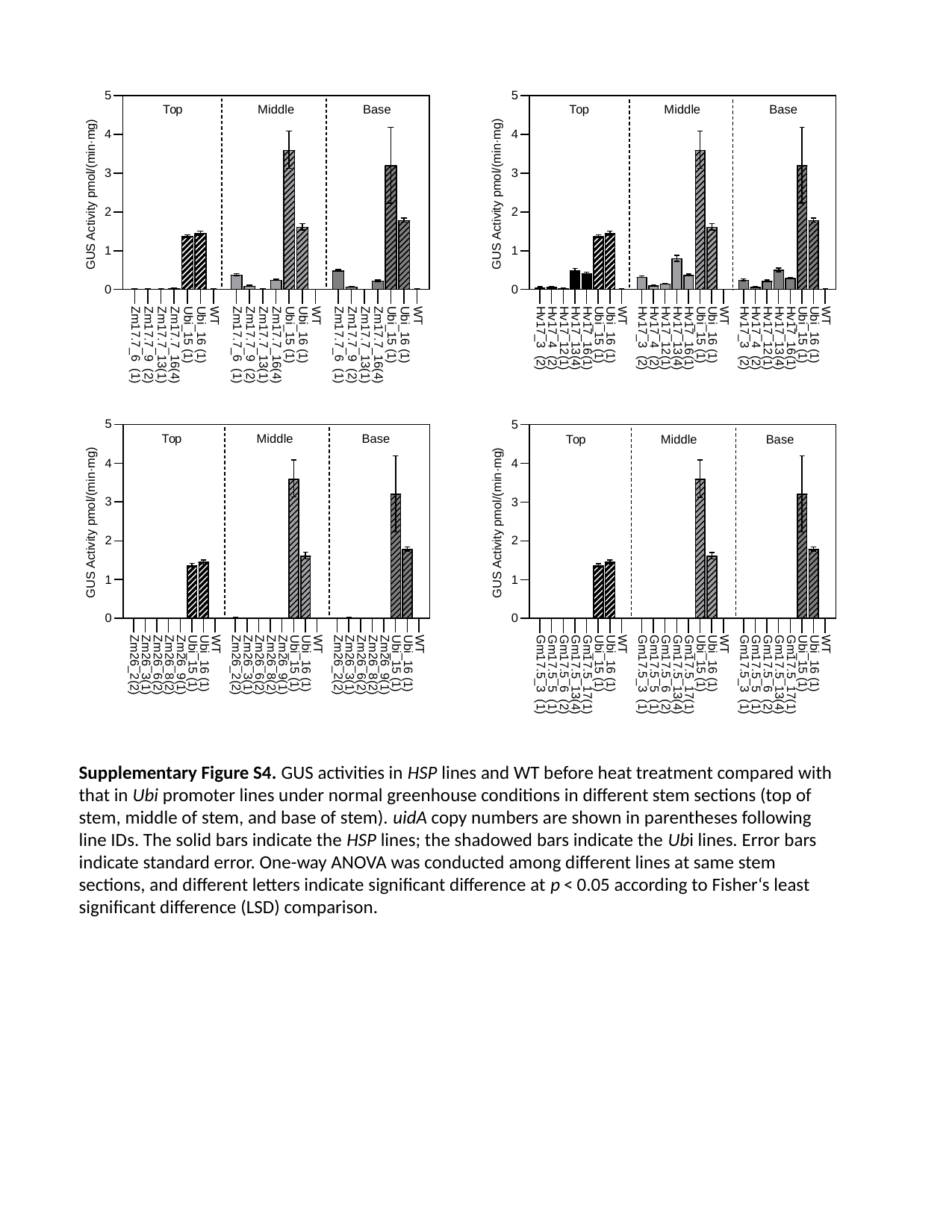

Supplementary Figure S4. GUS activities in HSP lines and WT before heat treatment compared with that in Ubi promoter lines under normal greenhouse conditions in different stem sections (top of stem, middle of stem, and base of stem). uidA copy numbers are shown in parentheses following line IDs. The solid bars indicate the HSP lines; the shadowed bars indicate the Ubi lines. Error bars indicate standard error. One-way ANOVA was conducted among different lines at same stem sections, and different letters indicate significant difference at p < 0.05 according to Fisher‘s least significant difference (LSD) comparison.

## Slide 5
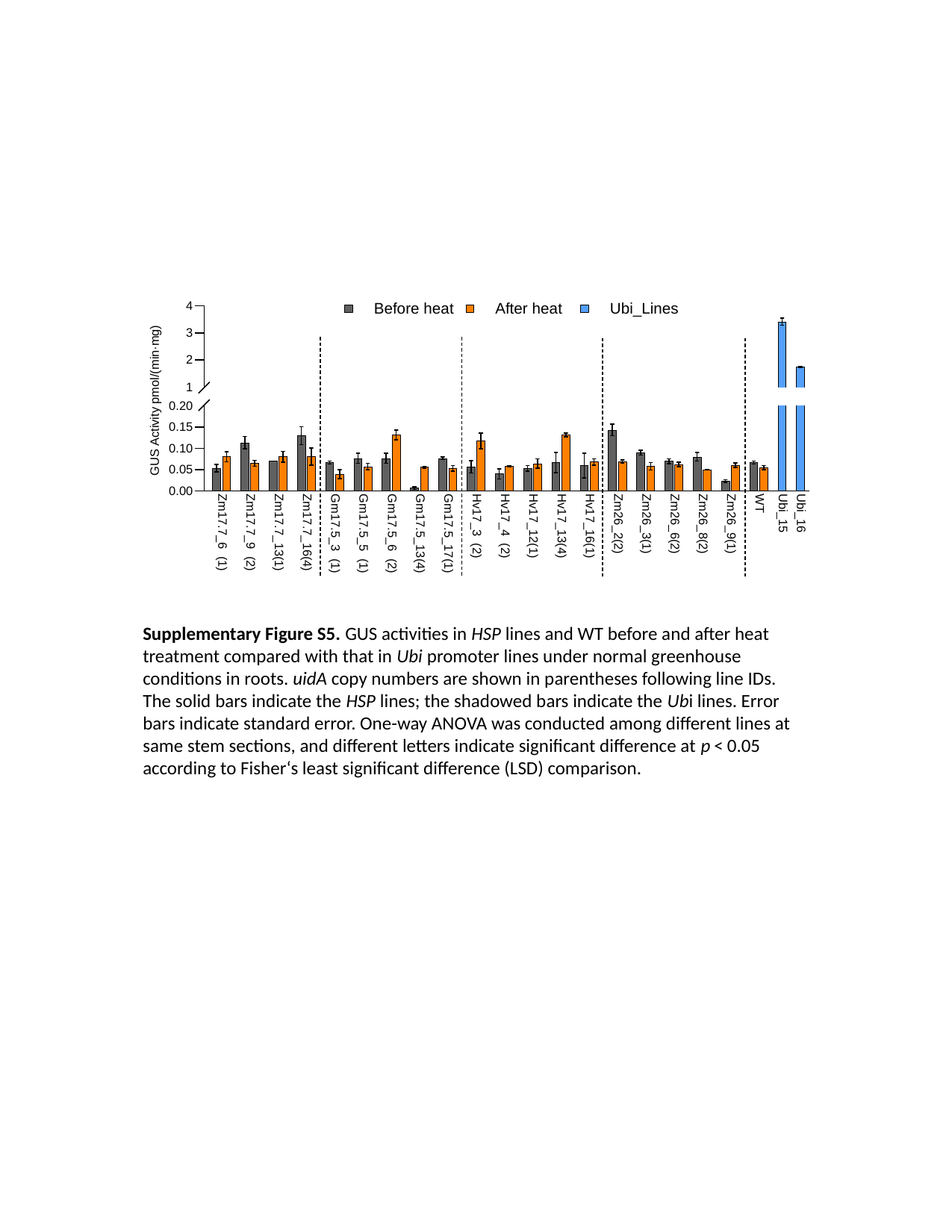

Supplementary Figure S5. GUS activities in HSP lines and WT before and after heat treatment compared with that in Ubi promoter lines under normal greenhouse conditions in roots. uidA copy numbers are shown in parentheses following line IDs. The solid bars indicate the HSP lines; the shadowed bars indicate the Ubi lines. Error bars indicate standard error. One-way ANOVA was conducted among different lines at same stem sections, and different letters indicate significant difference at p < 0.05 according to Fisher‘s least significant difference (LSD) comparison.

## Slide 6
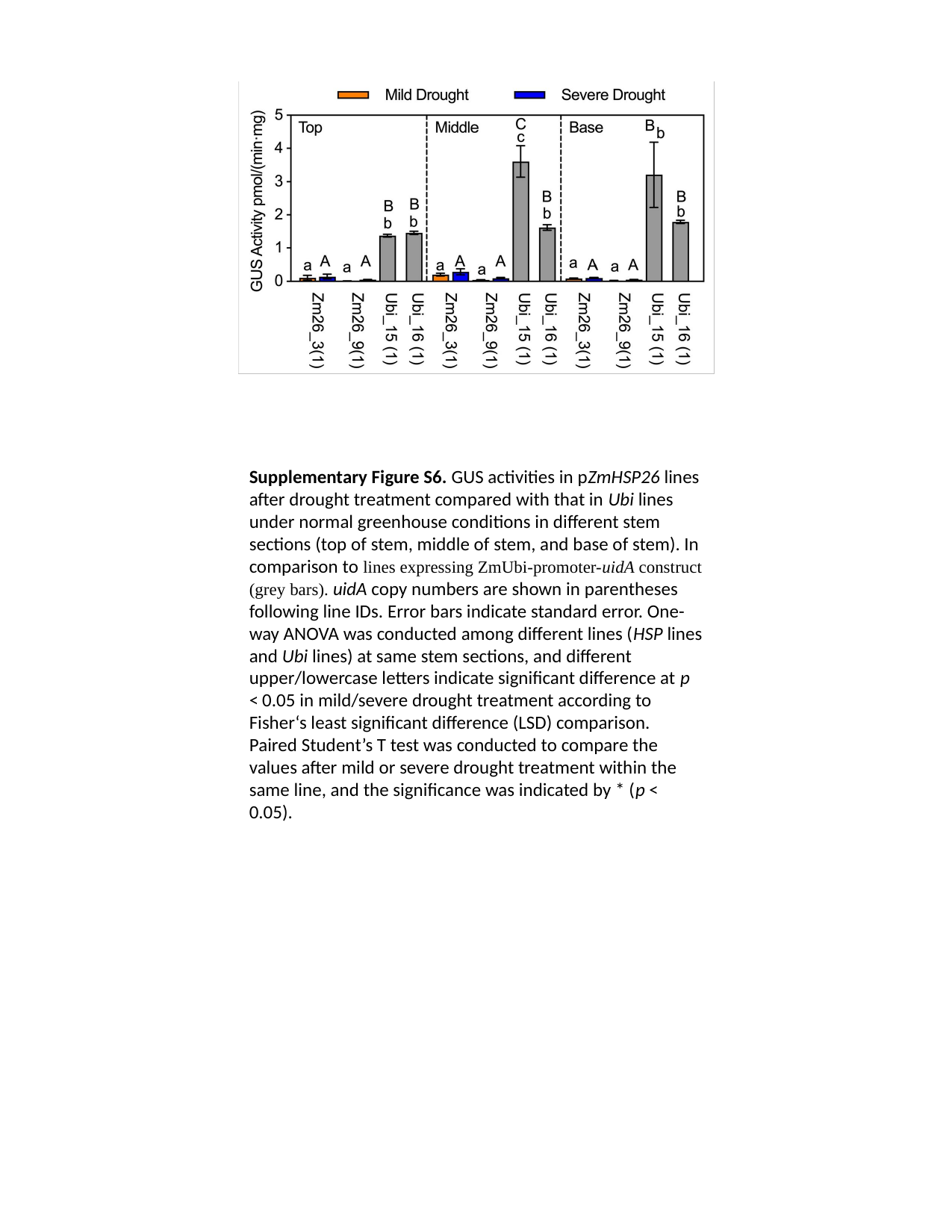

Supplementary Figure S6. GUS activities in pZmHSP26 lines after drought treatment compared with that in Ubi lines under normal greenhouse conditions in different stem sections (top of stem, middle of stem, and base of stem). In comparison to lines expressing ZmUbi-promoter-uidA construct (grey bars). uidA copy numbers are shown in parentheses following line IDs. Error bars indicate standard error. One-way ANOVA was conducted among different lines (HSP lines and Ubi lines) at same stem sections, and different upper/lowercase letters indicate significant difference at p < 0.05 in mild/severe drought treatment according to Fisher‘s least significant difference (LSD) comparison. Paired Student’s T test was conducted to compare the values after mild or severe drought treatment within the same line, and the significance was indicated by * (p < 0.05).

## Slide 7
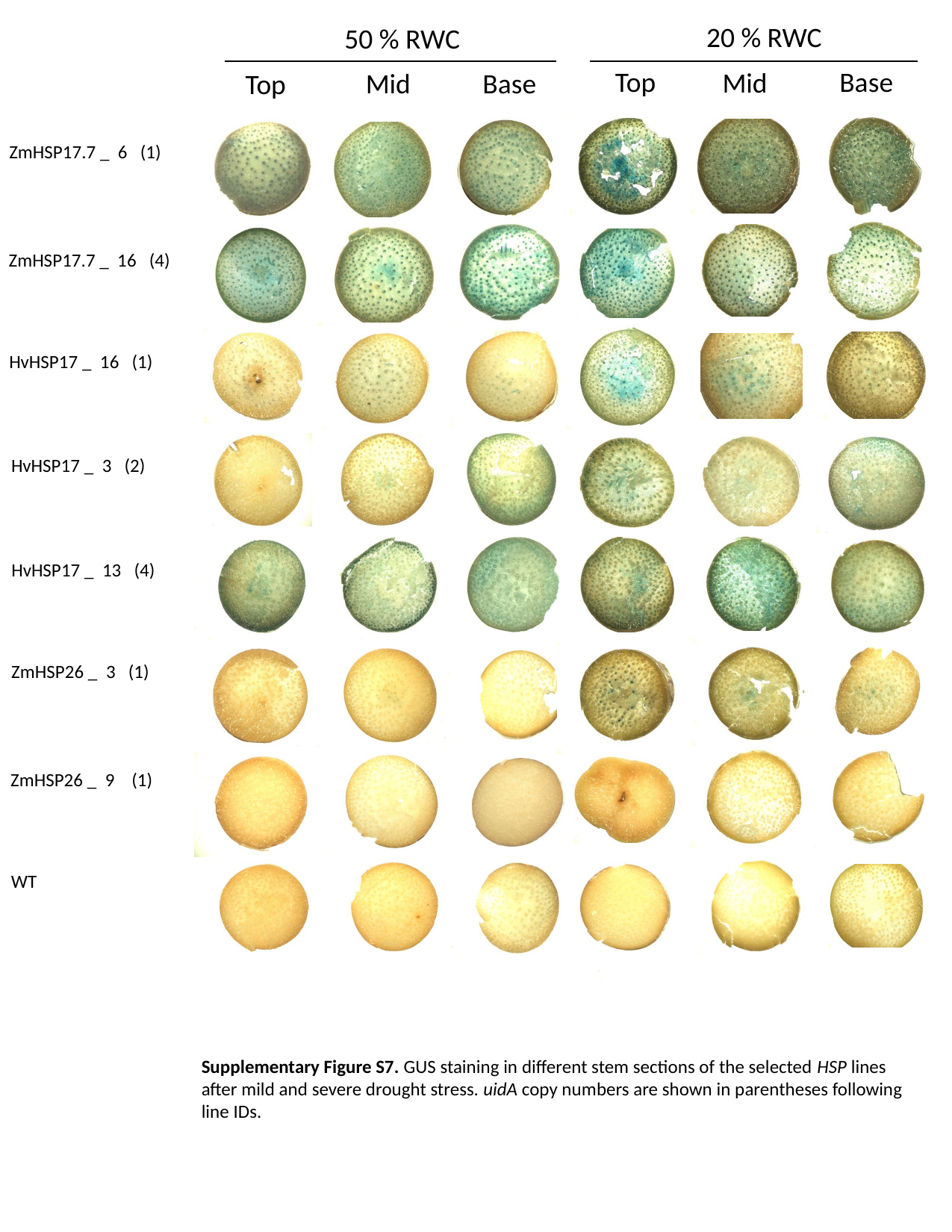

20 % RWC
50 % RWC
Top
ZmHSP17.7 _ 6 (1)
ZmHSP17.7 _ 16 (4)
HvHSP17 _ 16 (1)
HvHSP17 _ 3 (2)
HvHSP17 _ 13 (4)
ZmHSP26 _ 3 (1)
ZmHSP26 _ 9 (1)
WT
Top
Base
Mid
Base
Mid
Supplementary Figure S7. GUS staining in different stem sections of the selected HSP lines after mild and severe drought stress. uidA copy numbers are shown in parentheses following line IDs.

## Slide 8
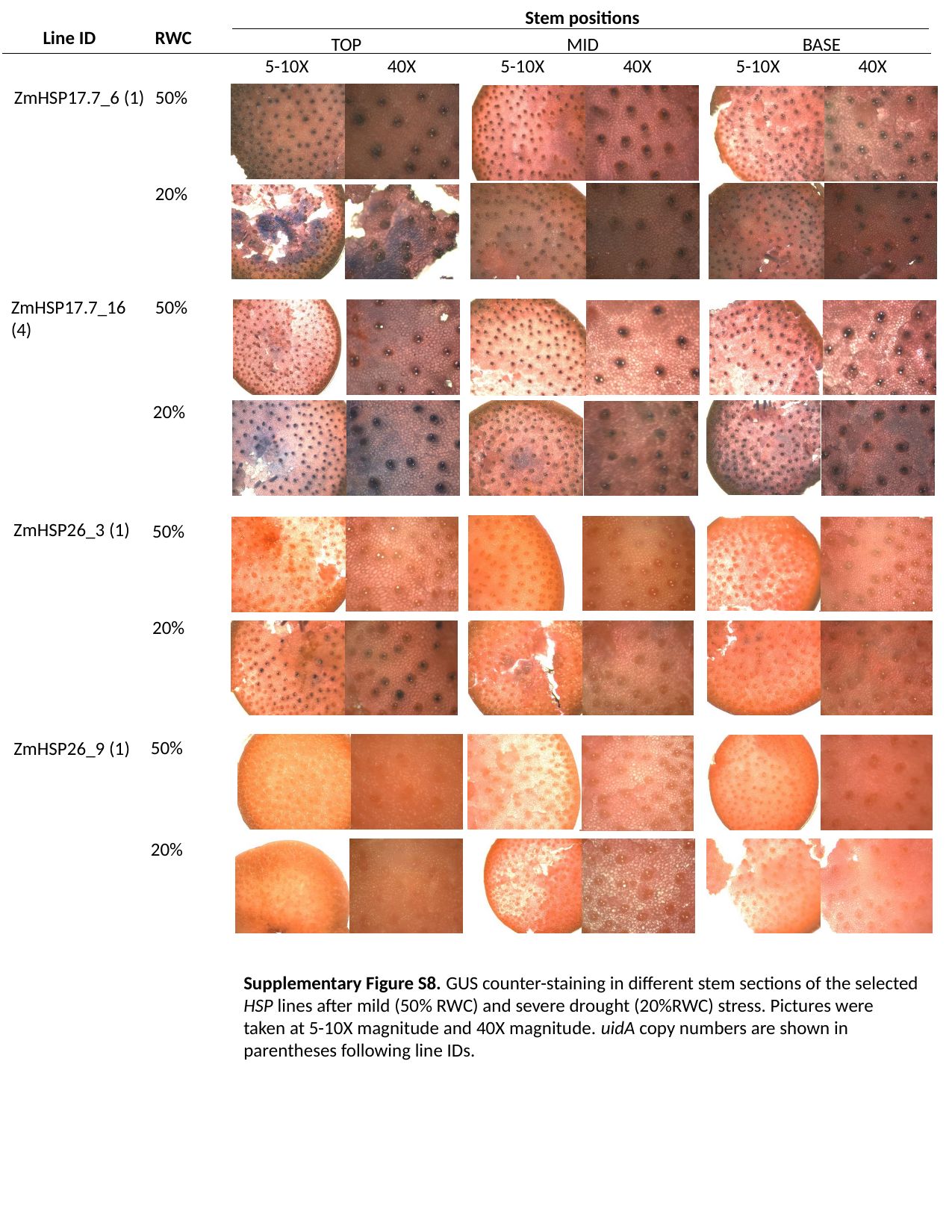

Stem positions
RWC
Line ID
TOP
MID
BASE
5-10X
40X
5-10X
40X
5-10X
40X
50%
20%
ZmHSP17.7_6 (1)
ZmHSP17.7_16 (4)
ZmHSP26_3 (1)
ZmHSP26_9 (1)
50%
20%
50%
20%
50%
20%
Supplementary Figure S8. GUS counter-staining in different stem sections of the selected HSP lines after mild (50% RWC) and severe drought (20%RWC) stress. Pictures were taken at 5-10X magnitude and 40X magnitude. uidA copy numbers are shown in parentheses following line IDs.

## Slide 9
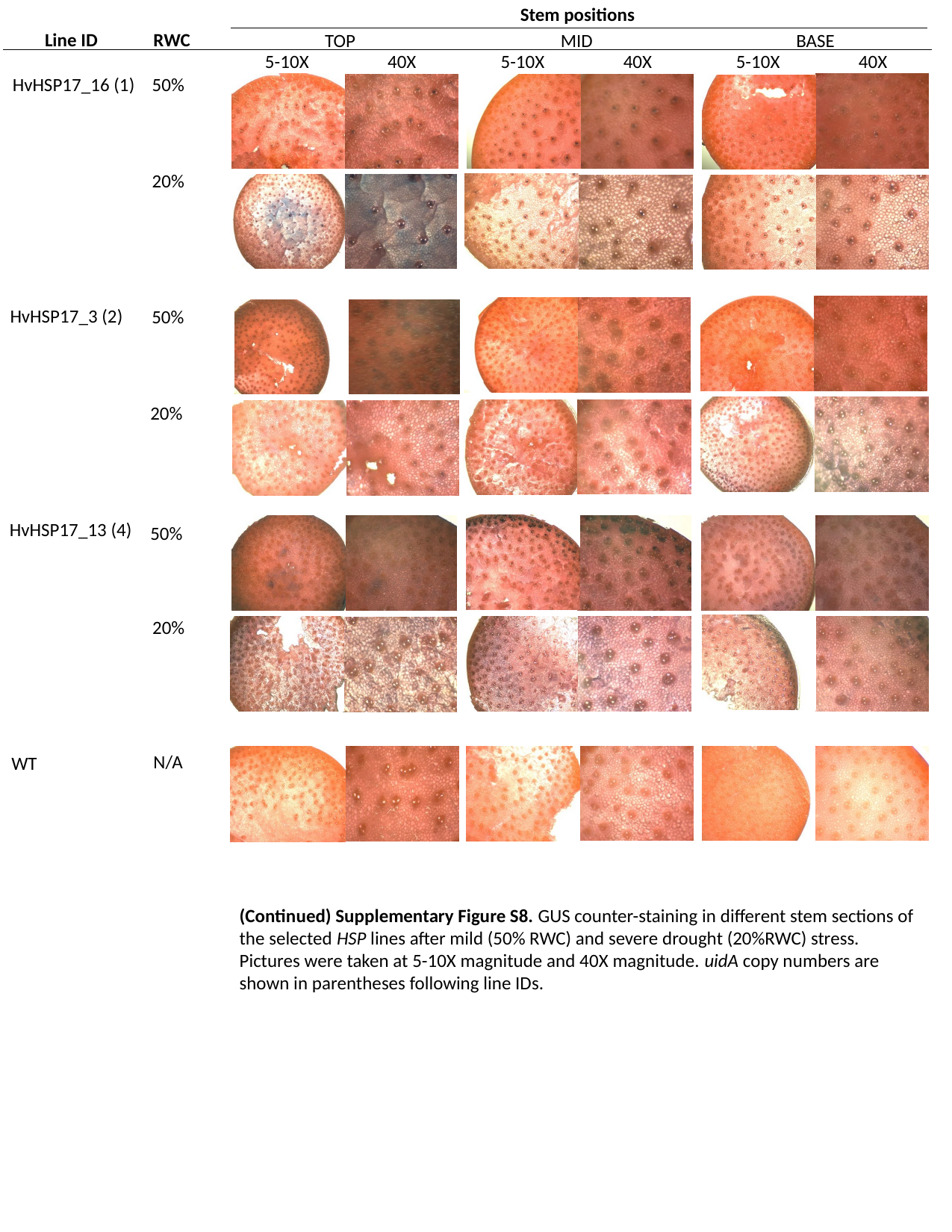

Stem positions
RWC
Line ID
TOP
MID
BASE
40X
5-10X
40X
5-10X
40X
5-10X
HvHSP17_16 (1)
HvHSP17_3 (2)
50%
20%
50%
20%
HvHSP17_13 (4)
WT
50%
20%
N/A
(Continued) Supplementary Figure S8. GUS counter-staining in different stem sections of the selected HSP lines after mild (50% RWC) and severe drought (20%RWC) stress. Pictures were taken at 5-10X magnitude and 40X magnitude. uidA copy numbers are shown in parentheses following line IDs.
