# Supplementary material for: Comparing four heat-inducible promoters in stably transformed sugarcane regarding spatial and temporal control of transgene expression reveals candidates to drive stem-preferred transgene expression
Source: Front Plant Sci. 2025 Dec 3;16:1709171. doi: 10.3389/fpls.2025.1709171 (PMC12708596; doi:10.3389/fpls.2025.1709171)
Supplement: Supplementary file 2 [file Table1.docx]

**Supplementary Table S1.** Primers used in PCR, *uidA* copy number assay, and qPCR.

| **Assay** | **Gene** | **Forward Primer (5’ to 3’)** | **Reverse Primer (5’ to 3’)** |
| --- | --- | --- | --- |
| PCR/ *uidA* copy number assay | *uidA* | GAGGTGTTCATCCCGAAGGG | ATGGTCTGCCAGTTCAGCTC |
| qPCR reaction | *GAPDH* | CACGGCCACTGGAAGCA | TCCTCAGGGTTCCTGATGCC |
|  | *uidA* | GTTCTACTTCACCGGCTTCG | CCCAGTCCAGCATCTCCTC |

**Supplementary Table S2.** Buffers used in GUS staining assay and MUG assay.

| **Buffer Name** | **Components** |
| --- | --- |
| GUS staining solution | 40 mM NaH_2_PO_4_  60 mM Na_2_HPO_4_  10 mM Na_2_EDTA  0.5 mM K_3_Fe(CN)_6_  0.5 mM K_4_Fe(CN)_6_.3H_2_O  0.1% (v/v) Triton  0.5 mg/mL 5-bromo-4-chloro-3-indolyl-β-D-glucuronic acid |
| GUS extraction buffer (GEB) | 28 mM Na_2_HPO_4_  20 mM NaH_2_PO_4_  10 mM Na_2_EDTA  0.1% (v/v) Triton  0.1% (v/v) SDS  0.07% (v/v) β-mercaptoethanol |
| Assay MUG buffer (AMB) | GEB supplemented with 0.5 mM 4-Methylumbelliferyl-β-D-glucuronide hydrate (MUG) |

**Supplementary Table S3**. Predicted GUS copy number of the transgenic HSP lines (V0)

| **Line ID** | **Predicted GUS Copy Number** |
| --- | --- |
| Gm17.5_1 | 1 |
| Gm17.5_3 | 1 |
| Gm17.5_5 | 1 |
| Gm17.5_6 | 2 |
| Gm17.5_9 | 1 |
| Gm17.5_12 | 1 |
| Gm17.5_13 | 4 |
| Gm17.5_17 | 1 |
| Hv17_2 | 2 |
| Hv17_3 | 2 |
| Hv17_4 | 2 |
| Hv17_12 | 1 |
| Hv17_13 | 4 |
| Hv17_14 | 5 |
| Hv17_15 | 5 |
| Hv17_16 | 1 |
| Zm26_1 | 4 |
| Zm26_2 | 2 |
| Zm26_3 | 1 |
| Zm26_6 | 2 |
| Zm26_7 | 2 |
| Zm26_8 | 2 |
| Zm26_9 | 1 |
| Zm26_12 | 2 |
| Zm26_14 | 2 |
| Zm17.7_2 | 4 |
| Zm17.7_4 | 4 |
| Zm17.7_6 | 1 |
| Zm17.7_9 | 2 |
| Zm17.7_13 | 1 |
| Zm17.7_16 | 4 |
| Zm17.7_19 | 4 |

**Supplementary Table S4a.** *Cis*-element call using PlantPAN (a), PlantCARE (b), and PlantTFDB (c).

| **PlantPAN3.0** | | | | | | | |
| --- | --- | --- | --- | --- | --- | --- | --- |
| Promoter | TF family | 0 to -100 bp | -100 to -300 bp | -300 to -600 bp | -600 to -1000 bp | upstream -1000 bp | Sum |
| pGmHSP17.5 | AP2 |  | 1 |  |  |  | 1 |
|  | AT-Hook |  | 4 | 16 |  |  | 20 |
|  | C2H2 |  |  | 3 |  |  | 3 |
|  | EIN3 |  | 2 |  |  |  | 2 |
|  | Homeodomain |  | 1 |  |  |  | 1 |
|  | MADF |  |  | 2 |  |  | 2 |
|  | MADS box |  | 1 |  |  |  | 1 |
|  | Myb/SANT | 7 | 4 | 8 |  |  | 19 |
|  | SBP |  | 15 |  |  |  | 15 |
|  | TBP |  | 5 | 2 |  |  | 7 |
|  | bHLH |  |  | 1 |  |  | 1 |
|  | bZIP |  | 2 |  |  |  | 2 |
| pHvHSP17.7 | AP2 | 3 |  |  |  |  | 3 |
|  | AP2; ERF | 1 |  |  |  |  | 1 |
|  | AT-Hook |  | 1 | 5 | 2 |  | 8 |
|  | C2H2 |  | 2 | 2 |  |  | 4 |
|  | EIN3 | 1 |  |  |  |  | 1 |
|  | GATA |  | 1 |  |  |  | 1 |
|  | Homeodomain | 2 |  | 18 |  |  | 20 |
|  | MADS box |  |  | 1 |  |  | 1 |
|  | Myb/SANT | 1 | 1 | 5 |  |  | 7 |
|  | NAC; NAM |  |  |  | 1 |  | 1 |
|  | Storekeeper |  |  |  | 1 |  | 1 |
|  | TBP |  |  | 8 | 7 |  | 15 |
|  | TCP |  |  | 1 |  |  | 1 |
|  | bHLH |  | 1 | 1 |  |  | 2 |
|  | bZIP |  | 3 | 2 | 3 |  | 8 |
| pZmHSP17.7 | AP2 |  |  |  | 52 | 2 | 54 |
|  | AP2; B3 |  |  | 2 |  | 1 | 3 |
|  | AP2; ERF |  |  |  | 2 |  | 2 |
|  | AT-Hook |  |  | 1 |  | 12 | 13 |
|  | B3 |  |  |  | 1 |  | 1 |
|  | C2H2 | 1 |  |  |  | 4 | 5 |
|  | CG-1 |  |  |  | 4 |  | 4 |
|  | E2F |  |  |  | 2 |  | 2 |
|  | EIN3 |  |  |  |  | 1 | 1 |
|  | GATA | 18 | 7 |  | 15 | 4 | 44 |
|  | Homeodomain | 7 |  | 6 | 2 | 1 | 16 |
|  | MADF |  |  |  | 1 | 2 | 3 |
|  | MADS box |  |  |  |  | 2 | 2 |
|  | Myb/SANT | 1 |  |  |  | 28 | 29 |
|  | NAC; NAM |  |  |  | 2 | 3 | 5 |
|  | Storekeeper |  |  |  |  | 1 | 1 |
|  | TBP |  | 3 |  |  | 2 | 5 |
|  | TCP |  | 1 | 1 | 1 | 1 | 4 |
|  | WRKY | 11 |  |  | 1 | 6 | 18 |
|  | bHLH |  | 56 | 5 | 4 | 3 | 68 |
|  | bZIP |  | 16 | 12 |  | 3 | 31 |
| pZmHSP26 | AP2 |  | 7 |  |  |  | 7 |
|  | AP2; ERF |  | 1 |  |  |  | 1 |
|  | AT-Hook |  |  | 1 | 2 |  | 3 |
|  | B3 |  | 1 |  | 2 |  | 3 |
|  | C2H2 |  |  | 2 |  |  | 2 |
|  | GATA | 19 |  |  | 2 |  | 21 |
|  | MADS box | 2 |  |  |  |  | 2 |
|  | Myb/SANT |  |  | 1 |  |  | 1 |
|  | NAC; NAM |  | 1 |  |  |  | 1 |
|  | TBP |  | 3 | 4 | 3 |  | 10 |
|  | TCP |  | 3 | 1 |  |  | 4 |
|  | WRKY |  | 7 |  |  |  | 7 |
|  | bHLH | 2 |  | 4 | 4 |  | 10 |
|  | bZIP |  | 4 | 3 | 3 |  | 10 |

**Supplementary Table S4b.** *Cis*-element call using PlantPAN (a), PlantCARE (b), and PlantTFDB (c).

| **PlantCARE** | | | | | | | |
| --- | --- | --- | --- | --- | --- | --- | --- |
| Promoter | TF family | 0 to -100 bp | -100 to -300 bp | -300 to -600 bp | -600 to -1000 bp | upstream -1000 bp | Sum |
| pGmHSP17.5 | AP2 |  | 1 |  |  |  | 1 |
|  | AT-Hook |  | 4 | 16 |  |  | 20 |
|  | C2H2 |  |  | 3 |  |  | 3 |
|  | EIN3 |  | 2 |  |  |  | 2 |
|  | Homeodomain |  | 1 |  |  |  | 1 |
|  | MADF |  |  | 2 |  |  | 2 |
|  | MADS box |  | 1 |  |  |  | 1 |
|  | Myb/SANT | 7 | 4 | 8 |  |  | 19 |
|  | SBP |  | 15 |  |  |  | 15 |
|  | TBP |  | 5 | 2 |  |  | 7 |
|  | bHLH |  |  | 1 |  |  | 1 |
|  | bZIP |  | 2 |  |  |  | 2 |
| pHvHSP17.7 | AP2 | 3 |  |  |  |  | 3 |
|  | AP2; ERF | 1 |  |  |  |  | 1 |
|  | AT-Hook |  | 1 | 5 | 2 |  | 8 |
|  | C2H2 |  | 2 | 2 |  |  | 4 |
|  | EIN3 | 1 |  |  |  |  | 1 |
|  | GATA |  | 1 |  |  |  | 1 |
|  | Homeodomain | 2 |  | 18 |  |  | 20 |
|  | MADS box |  |  | 1 |  |  | 1 |
|  | Myb/SANT | 1 | 1 | 5 |  |  | 16 |
|  | NAC; NAM |  |  |  | 1 |  | 1 |
|  | Storekeeper |  |  |  | 1 |  | 1 |
|  | TBP |  |  | 8 | 7 |  | 15 |
|  | TCP |  |  | 1 |  |  | 10 |
|  | bHLH |  | 1 | 1 |  |  | 2 |
|  | bZIP |  | 3 | 2 | 3 |  | 8 |
| pZmHSP17.7 | AP2 |  |  |  | 52 | 2 | 54 |
|  | AP2; B3 |  |  | 2 |  | 1 | 3 |
|  | AP2; ERF |  |  |  | 2 |  | 2 |
|  | AT-Hook |  |  | 1 |  | 12 | 13 |
|  | B3 |  |  |  | 1 |  | 1 |
|  | C2H2 | 1 |  |  |  | 4 | 5 |
|  | CG-1 |  |  |  | 4 |  | 4 |
|  | E2F |  |  |  | 2 |  | 2 |
|  | EIN3 |  |  |  |  | 1 | 1 |
|  | GATA | 18 | 7 |  | 15 | 4 | 44 |
|  | Homeodomain | 7 |  | 6 | 2 | 1 | 25 |
|  | MADF |  |  |  | 1 | 2 | 3 |
|  | MADS box |  |  |  |  | 2 | 2 |
|  | Myb/SANT | 1 |  |  |  | 28 | 29 |
|  | NAC; NAM |  |  |  | 2 | 3 | 5 |
|  | Storekeeper |  |  |  |  | 1 | 1 |
|  | TBP |  | 3 |  |  | 2 | 5 |
|  | TCP |  | 1 | 1 | 1 | 1 | 13 |
|  | WRKY | 11 |  |  | 1 | 6 | 18 |
|  | bHLH |  | 56 | 5 | 4 | 3 | 68 |
|  | bZIP |  | 16 | 12 |  | 3 | 31 |
| pZmHSP26 | AP2 |  | 7 |  |  |  | 7 |
|  | AP2; ERF |  | 1 |  |  |  | 1 |
|  | AT-Hook |  |  | 1 | 2 |  | 3 |
|  | B3 |  | 1 |  | 2 |  | 3 |
|  | C2H2 |  |  | 2 |  |  | 2 |
|  | GATA | 19 |  |  | 2 |  | 21 |
|  | MADS box | 2 |  |  |  |  | 2 |
|  | Myb/SANT |  |  | 1 |  |  | 1 |
|  | NAC; NAM |  | 1 |  |  |  | 1 |
|  | TBP |  | 3 | 4 | 3 |  | 10 |
|  | TCP |  | 3 | 1 |  |  | 4 |
|  | WRKY |  | 7 |  |  |  | 7 |
|  | bHLH | 2 |  | 4 | 4 |  | 10 |
|  | bZIP |  | 4 | 3 | 3 |  | 10 |

**Supplementary Table S4c.** *Cis*-element call using PlantPAN (a), PlantCARE (b), and PlantTFDB (c).

| **PlantTFDB** | | | | | | | |
| --- | --- | --- | --- | --- | --- | --- | --- |
| Promoter | TF family | 0 to -100 bp | -100 to -300 bp | -300 to -600 bp | -600 to -1000 bp | upstream -1000 bp | Sum |
| pGmHSP17.5 | ARR-B | 1 |  |  |  |  | 1 |
|  | B3 |  | 2 |  |  |  | 2 |
|  | C2H2 | 4 |  | 2 |  |  | 6 |
|  | Dof | 8 |  |  |  |  | 8 |
|  | GATA |  | 2 |  |  |  | 2 |
|  | GRAS | 1 |  |  |  |  | 1 |
|  | HSF | 15 | 8 |  |  |  | 23 |
|  | MIKC_MADS |  | 14 | 2 |  |  | 16 |
|  | MYB | 1 |  |  |  |  | 1 |
|  | MYB_related | 1 |  | 9 |  |  | 10 |
|  | NAC | 1 |  |  |  |  | 1 |
|  | S1Fa-like | 1 | 1 |  |  |  | 2 |
|  | Trihelix |  |  | 3 |  |  | 3 |
|  | bZIP |  | 1 |  |  |  | 1 |
| pHvHSP17.7 | AP2 |  | 5 | 1 |  |  | 6 |
|  | ARF |  |  |  | 2 |  | 2 |
|  | B3 |  | 1 | 3 |  |  | 4 |
|  | BBR-BPC |  | 7 |  |  |  | 7 |
|  | BES1 |  | 1 |  |  |  | 1 |
|  | C2H2 |  |  | 2 | 2 |  | 4 |
|  | C3H |  |  | 1 |  |  | 1 |
|  | Dof |  | 1 | 12 |  |  | 13 |
|  | E2F/DP |  |  | 5 |  |  | 5 |
|  | EIL |  |  | 1 |  |  | 1 |
|  | ERF | 9 | 3 | 9 |  |  | 21 |
|  | FAR1 |  |  |  | 1 |  | 1 |
|  | G2-like |  |  |  | 2 |  | 2 |
|  | GATA |  | 1 |  |  |  | 1 |
|  | GRAS |  | 1 |  |  |  | 1 |
|  | HD-ZIP |  |  | 2 |  |  | 2 |
|  | HSF |  | 7 | 1 |  |  | 8 |
|  | MIKC_MADS | 1 | 1 | 1 |  |  | 3 |
|  | MYB | 8 | 1 | 2 | 7 |  | 18 |
|  | MYB_related |  |  | 2 |  |  | 2 |
|  | NAC |  | 1 | 3 |  |  | 4 |
|  | Nin-like |  |  | 1 |  |  | 1 |
|  | S1Fa-like |  | 1 |  |  |  | 1 |
|  | SBP |  |  | 1 |  |  | 1 |
|  | TCP |  | 2 | 11 |  |  | 13 |
|  | Trihelix |  | 1 |  |  |  | 1 |
|  | WOX |  |  |  | 1 |  | 1 |
|  | ZF-HD |  |  | 1 |  |  | 1 |
|  | bZIP | 2 | 4 |  |  |  | 6 |
| pZmHSP17.7 | AP2 |  | 3 | 2 | 1 | 1 | 7 |
|  | ARF |  |  |  | 4 | 4 | 8 |
|  | ARR-B | 1 |  |  |  | 5 | 6 |
|  | B3 |  | 1 | 2 | 2 | 4 | 9 |
|  | BBR-BPC |  | 35 | 2 |  | 15 | 52 |
|  | BES1 |  | 5 |  | 2 | 5 | 12 |
|  | C2H2 | 1 | 8 | 9 | 3 | 1 | 22 |
|  | CAMTA |  |  | 1 | 7 | 1 | 9 |
|  | CPP |  |  |  |  | 1 | 1 |
|  | Dof |  |  |  |  | 6 | 6 |
|  | E2F/DP |  | 1 |  | 2 | 3 | 6 |
|  | EIL |  |  | 1 |  | 1 | 2 |
|  | ERF |  | 49 | 12 | 568 | 26 | 655 |
|  | FAR1 |  | 1 |  | 1 |  | 2 |
|  | G2-like | 1 |  |  |  | 4 | 5 |
|  | GATA | 2 |  | 2 | 4 | 9 | 17 |
|  | GRAS |  | 2 | 2 |  | 4 | 8 |
|  | GRF |  |  | 1 |  |  | 1 |
|  | GeBP |  |  |  | 5 | 1 | 6 |
|  | HD-ZIP |  |  | 1 |  | 1 | 2 |
|  | HSF |  | 12 |  |  |  | 12 |
|  | LBD |  |  | 1 | 52 | 5 | 58 |

**Supplementary Table S4c.** *Cis*-element call using PlantPAN (a), PlantCARE (b), or PlantTFDB (c). (Continued)

| **PlantTFDB** | | | | | | | |
| --- | --- | --- | --- | --- | --- | --- | --- |
| pZmHSP17.7 | TF family | 0 to -100 bp | -100 to -300 bp | -300 to -600 bp | -600 to -1000 bp | upstream -1000 bp | Sum |
|  | LBD |  |  | 1 | 52 | 5 | 58 |
|  | MIKC_MADS |  | 2 |  |  | 11 | 13 |
|  | MYB | 2 | 1 | 4 | 12 | 3 | 22 |
|  | MYB_related |  |  |  | 1 | 1 | 2 |
|  | NAC | 3 | 2 |  | 8 | 4 | 17 |
|  | Nin-like |  |  |  | 3 | 4 | 7 |
|  | S1Fa-like |  | 2 |  |  |  | 2 |
|  | SBP |  |  |  | 1 | 1 | 2 |
|  | TCP |  |  |  | 26 | 8 | 34 |
|  | Trihelix |  |  | 1 | 7 | 4 | 12 |
|  | VOZ |  |  |  | 1 |  | 1 |
|  | WRKY |  |  |  | 1 | 3 | 4 |
|  | bHLH |  | 28 | 9 | 12 | 4 | 53 |
|  | bZIP |  | 12 | 2 | 12 | 3 | 29 |
| pZmHSP26 | AP2 |  | 2 |  | 1 |  | 3 |
|  | ARF |  | 5 |  | 3 |  | 8 |
|  | ARR-B | 1 |  |  |  |  | 1 |
|  | B3 |  |  |  | 2 |  | 2 |
|  | BBR-BPC | 3 |  |  | 4 |  | 7 |
|  | BES1 |  |  | 2 |  |  | 2 |
|  | C2H2 | 1 | 8 |  | 6 |  | 15 |
|  | CAMTA |  |  | 1 |  |  | 1 |
|  | E2F/DP |  |  | 1 | 1 |  | 2 |
|  | ERF | 2 | 122 | 35 | 67 |  | 226 |
|  | G2-like | 1 |  | 2 |  |  | 3 |
|  | GATA | 8 |  |  |  |  | 8 |
|  | GRAS |  | 1 |  | 1 |  | 2 |
|  | GeBP |  |  |  | 1 |  | 1 |
|  | HSF |  | 7 | 9 |  |  | 16 |
|  | LBD |  | 4 | 1 | 11 |  | 16 |
|  | MIKC_MADS | 3 | 1 |  |  |  | 4 |
|  | MYB | 4 | 1 | 7 | 3 |  | 15 |
|  | NAC |  | 1 | 5 | 3 |  | 9 |
|  | Nin-like |  | 2 |  | 2 |  | 4 |
|  | S1Fa-like |  | 2 |  |  |  | 2 |
|  | SRS |  |  |  | 2 |  | 2 |
|  | TCP |  | 9 | 2 | 4 |  | 15 |
|  | Trihelix |  | 1 | 1 | 2 |  | 4 |
|  | WRKY |  | 1 |  |  |  | 1 |
|  | bHLH |  | 1 | 1 | 8 |  | 10 |
|  | bZIP |  |  | 6 | 3 |  | 9 |

**Supplementary Table S5a.** Customized HSE motif call using Python code (<https://github.com/qiandemoni/HSE_sequence_finder>) for *pHvHSP17.7* (a), *pGmHSP17.5* (b), *pZmHSP17.7* (c), and *pZmHSP26* (d).

| **Sequence ID** | **Found Subsequence (mismatch in red)** | **Start Position** | **Transcript Start Site Position** | **Mismatch Count** | **Insertion Size** | **Pattern** | **Ranking** |
| --- | --- | --- | --- | --- | --- | --- | --- |
| pHvHSP17.7 | ATTCTGGAAA | 303 | -425 | 0 | 0 | nTTCn-nGAAn | 1 |
|  | ATTCTTGAAT | 327 | -401 | 0 | 0 | nTTCn-nGAAn | 1 |
|  | ATTCTGGAAACGGCC | 303 | -425 | 2 | 0 | nTTCn-nGAAn-nTTCn | 1 |
|  | ATTCTTGAATCTAGA | 327 | -401 | 2 | 0 | nTTCn-nGAAn-nTTCn | 1 |
|  | AGAATTTTAA | 74 | -654 | 1 | 0 | nGAAn-nTTCn | 2A |
|  | TGAACTTTAT | 158 | -570 | 1 | 0 | nGAAn-nTTCn | 2A |
|  | AGAAAAATCT | 379 | -349 | 1 | 0 | nGAAn-nTTCn | 2A |
|  | GGCAGGTTCG | 435 | -293 | 1 | 0 | nGAAn-nTTCn | 2A |
|  | AGACCCTTCG | 465 | -263 | 1 | 0 | nGAAn-nTTCn | 2A |
|  | AGATTCTTCA | 562 | -166 | 1 | 0 | nGAAn-nTTCn | 2A |
|  | TTGCTGGAAT | 354 | -374 | 1 | 0 | nTTCn-nGAAn | 2A |
|  | TTTATAGAAA | 374 | -354 | 1 | 0 | nTTCn-nGAAn | 2A |
|  | GTTCGTGAGT | 440 | -288 | 1 | 0 | nTTCn-nGAAn | 2A |
|  | ATTCTTCAAG | 564 | -164 | 1 | 0 | nTTCn-nGAAn | 2A |
|  | CTTCAAGATC | 567 | -161 | 1 | 0 | nTTCn-nGAAn | 2A |
|  | GGCAGGTTCGTGAGT | 435 | -293 | 2 | 0 | nGAAn-nTTCn-nGAAn | 2A |
|  | AGATTCTTCAAGATC | 562 | -166 | 2 | 0 | nGAAn-nTTCn-nGAAn | 2A |
|  | TTTATAGAAAAATCT | 374 | -354 | 2 | 0 | nTTCn-nGAAn-nTTCn | 2A |
|  | GGAATTATACA | 359 | -369 | 1 | 1 | nGAAn-nTTCn | 3 |
|  | CGAATGGCTCA | 496 | -232 | 1 | 1 | nGAAn-nTTCn | 3 |
|  | ATTCTGGAAAC | 303 | -425 | 1 | 1 | nTTCn-nGAAn | 3 |
|  | CTTGCTGGAAT | 353 | -375 | 1 | 1 | nTTCn-nGAAn | 3 |
|  | ATTTATAGAAA | 373 | -355 | 1 | 1 | nTTCn-nGAAn | 3 |
|  | CTACCTGGAAA | 511 | -217 | 1 | 1 | nTTCn-nGAAn | 3 |
|  | CTTGCTGGAATTATACA | 353 | -375 | 2 | 1 | nTTCn-nGAAn-nTTCn | 3 |
|  | AGTACTCTTTCT | 128 | -600 | 1 | 2 | nGAAn-nTTCn | 3 |
|  | AGAAAAATCTCA | 379 | -349 | 1 | 2 | nGAAn-nTTCn | 3 |
|  | CGAGATTCTTCA | 560 | -168 | 1 | 2 | nGAAn-nTTCn | 3 |
|  | CGAACAACATCC | 653 | -75 | 1 | 2 | nGAAn-nTTCn | 3 |
|  | AGATTAATTTCA | 682 | -46 | 1 | 2 | nGAAn-nTTCn | 3 |
|  | TTTCATTTGTAT | 5 | -723 | 1 | 2 | nTTCn-nGAAn | 3 |
|  | GTTGTAGAGAAT | 67 | -661 | 1 | 2 | nTTCn-nGAAn | 3 |
|  | GTCCCCTGGAAG | 448 | -280 | 1 | 2 | nTTCn-nGAAn | 3 |
|  | AGAAAAATCTCACAAAAAA | 379 | -349 | 2 | 2 | nGAAn-nTTCn-nGAAn | 3 |
|  | ATTGTGTAGTACTCTTTCT | 121 | -607 | 2 | 2 | nTTCn-nGAAn-nTTCn | 3 |
|  | CCTCGAGATTCTTCA | 557 | -171 | 2 | 0 | nTTCn-nGAAn-nTTCn | 4A |
|  | ACAATTATTTATAGAAA | 367 | -361 | 2 | 1 | nGAAn-nTTCn-nGAAn | 4B |
|  | ACAACCCATTCT | 320 | -408 | 1 | 2 | nGAAn-nTTCn | 4B |

Ranking score is based on the conservation level of imperfect HSE motifs as detailed below:

**1:** very likely to retain function (mildly reduced affinity/induction): no substitutions at conserved GAA or TTC positions of 2 pentamers (nGAAnnTTCn or nTTCnnGAAn) and may have a third pentamer with substitutions. No insertions between pentamers.

**2A:** likely to retain partial function (reduced affinity/induction): substitutions within a GAA where the substituted base is an *A* (i.e., one of the A’s but not the G) or single substitutions within a TTC where the substituted base is T (position 2) or C (position 3) with no insertions between pentamers.

**2B:** likely to retain partial function (reduced affinity/induction): no substitutions at conserved GAA or TTC positions of 2 pentamers (nGAAnnTTCn or nTTCnnGAAn) and may have a third pentamer with substitutions. 1-2 bp Insertions between pentamers.

**3:** less likely to retain partial function (more reduced affinity/induction): substitutions within a GAA where the substituted base is an *A* (i.e., one of the A’s but not the G) or single substitutions within a TTC where the substituted base is T (position 2) or C (position 3). 1-2 bp insertions between pentamers.

**4A:** least likely to retain partial function (most reduced affinity/induction): substitutions of the conserved G within a GAA (G→X at positions 2) or substitutions of the T in the TTC core (T→X at positions 2), without insertions between pentamers.

**4B:** least likely to retain partial function (most reduced affinity/induction): substitutions of the conserved G within a GAA (G→X at positions 2) or substitutions of the T in the TTC core (T→X at positions 2), with 1-2 bp insertions between pentamers.

**Supplementary Table S5b.** HSE motifs call using Python code (<https://github.com/qiandemoni/HSE_sequence_finder>) for *pHvHSP17.7* (a), *pGmHSP17.5* (b), *pZmHSP17.7* (c), and *pZmHSP26* (d).

| **Sequence ID** | **Found Subsequence (mismatch in red)** | **Start Position** | **Transcript Start Site Position** | **Mismatch Count** | **Insertion Size** | **Pattern** | **Ranking** |
| --- | --- | --- | --- | --- | --- | --- | --- |
| pGmHSP17.5 | AGAACCTTCG | 315 | -167 | 0 | 0 | nGAAn-nTTCn | 1 |
|  | TTTCTGGAAC | 406 | -76 | 0 | 0 | nTTCn-nGAAn | 1 |
|  | GGATTTTTCTGGAAC | 401 | -81 | 1 | 0 | nGAAn-nTTCn-nGAAn | 1 |
|  | TTTCTGGAACATACA | 406 | -76 | 1 | 0 | nTTCn-nGAAn-nTTCn | 1 |
|  | AGAACCTTCGTACAT | 315 | -167 | 2 | 0 | nGAAn-nTTCn-nGAAn | 1 |
|  | AGAAAGTGCA | 163 | -319 | 1 | 0 | nGAAn-nTTCn | 2A |
|  | GGAGAATTCC | 335 | -147 | 1 | 0 | nGAAn-nTTCn | 2A |
|  | GGAATCATCT | 357 | -125 | 1 | 0 | nGAAn-nTTCn | 2A |
|  | GGATTTTTCT | 401 | -81 | 1 | 0 | nGAAn-nTTCn | 2A |
|  | GGAACATACA | 411 | -71 | 1 | 0 | nGAAn-nTTCn | 2A |
|  | CTGCTTGAAA | 37 | -445 | 1 | 0 | nTTCn-nGAAn | 2A |
|  | TTTCAAAAAG | 71 | -411 | 1 | 0 | nTTCn-nGAAn | 2A |
|  | TTTTTTGAAA | 83 | -399 | 1 | 0 | nTTCn-nGAAn | 2A |
|  | ATTCTAAAAA | 104 | -378 | 1 | 0 | nTTCn-nGAAn | 2A |
|  | ATTCTGGACA | 133 | -349 | 1 | 0 | nTTCn-nGAAn | 2A |
|  | ATTTCAGAAA | 158 | -324 | 1 | 0 | nTTCn-nGAAn | 2A |
|  | TTTCAGAAAG | 159 | -323 | 1 | 0 | nTTCn-nGAAn | 2A |
|  | ATTTATGAAT | 257 | -225 | 1 | 0 | nTTCn-nGAAn | 2A |
|  | ATTCCAGACG | 340 | -142 | 1 | 0 | nTTCn-nGAAn | 2A |
|  | TTTATGGAAT | 352 | -130 | 1 | 0 | nTTCn-nGAAn | 2A |
|  | GGAGAATTCCAGACG | 335 | -147 | 2 | 0 | nGAAn-nTTCn-nGAAn | 2A |
|  | GGAATCATCTGAAAC | 357 | -125 | 2 | 0 | nGAAn-nTTCn-nGAAn | 2A |
|  | GGAACATACAAGATT | 411 | -71 | 2 | 0 | nGAAn-nTTCn-nGAAn | 2A |
|  | CTGCTTGAAAATTTT | 37 | -445 | 2 | 0 | nTTCn-nGAAn-nTTCn | 2A |
|  | TTTCTTAACATTTCA | 61 | -421 | 2 | 0 | nTTCn-nGAAn-nTTCn | 2A |
|  | ATTCTAAAAAATACT | 104 | -378 | 2 | 0 | nTTCn-nGAAn-nTTCn | 2A |
|  | ATTTCAGAAAGTGCA | 158 | -324 | 2 | 0 | nTTCn-nGAAn-nTTCn | 2A |
|  | ATTCCAGACGTTTTT | 340 | -142 | 2 | 0 | nTTCn-nGAAn-nTTCn | 2A |
|  | TTTATGGAATCATCT | 352 | -130 | 2 | 0 | nTTCn-nGAAn-nTTCn | 2A |
|  | TGAAAATTTT | 42 | -440 | 1 | 0 | nGAAn-nTTCn | 2A |
|  | TGAAAATTTTT | 42 | -440 | 1 | 1 | nGAAn-nTTCn | 3 |
|  | TGAAACATTGT | 191 | -291 | 1 | 1 | nGAAn-nTTCn | 3 |
|  | TGGAGAATTCC | 334 | -148 | 1 | 1 | nGAAn-nTTCn | 3 |
|  | AGGATTTTTCT | 400 | -82 | 1 | 1 | nGAAn-nTTCn | 3 |
|  | ATTTTTTGAAA | 82 | -400 | 1 | 1 | nTTCn-nGAAn | 3 |
|  | ATTCTAAAAAA | 104 | -378 | 1 | 1 | nTTCn-nGAAn | 3 |
|  | TTTTATGGAAT | 351 | -131 | 1 | 1 | nTTCn-nGAAn | 3 |
|  | ATTCAACCAAA | 378 | -104 | 1 | 1 | nTTCn-nGAAn | 3 |
|  | TTTTCTGGAAC | 405 | -77 | 1 | 1 | nTTCn-nGAAn | 3 |
|  | CTTCCTTTAAA | 435 | -47 | 1 | 1 | nTTCn-nGAAn | 3 |
|  | TGAAAATTTTTTAGATA | 42 | -440 | 2 | 1 | nGAAn-nTTCn-nGAAn | 3 |
|  | TGAATGATGTCCAGAAG | 262 | -220 | 2 | 1 | nGAAn-nTTCn-nGAAn | 3 |
|  | AGACGTTTTTATGGAAT | 345 | -137 | 2 | 1 | nGAAn-nTTCn-nGAAn | 3 |
|  | TTTTTCTTAACATTTCA | 59 | -423 | 2 | 1 | nTTCn-nGAAn-nTTCn | 3 |
|  | ATTCTAAAAAATACTTTCC | 104 | -378 | 1 | 2 | nTTCn-nGAAn-nTTCn | 3 |
|  | TGAAAATTTTTT | 42 | -440 | 1 | 2 | nGAAn-nTTCn | 3 |
|  | AGATATTTTTCT | 54 | -428 | 1 | 2 | nGAAn-nTTCn | 3 |
|  | TGAATGATGTCC | 262 | -220 | 1 | 2 | nGAAn-nTTCn | 3 |
|  | ATTCTAAAAAAT | 104 | -378 | 1 | 2 | nTTCn-nGAAn | 3 |
|  | TTTTTATGGAAT | 350 | -132 | 1 | 2 | nTTCn-nGAAn | 3 |
|  | ATTCAACCAAAT | 378 | -104 | 1 | 2 | nTTCn-nGAAn | 3 |
|  | TTTTTCTGGAAC | 404 | -78 | 1 | 2 | nTTCn-nGAAn | 3 |
|  | CTTCCTTTAAAT | 435 | -47 | 1 | 2 | nTTCn-nGAAn | 3 |
|  | AGTAGGATTTTTCTGGAAC | 397 | -85 | 2 | 2 | nGAAn-nTTCn-nGAAn | 3 |
|  | ATTTTTTAGATATTTTTCT | 47 | -435 | 2 | 2 | nTTCn-nGAAn-nTTCn | 3 |
|  | ACAATATTCT | 99 | -383 | 1 | 0 | nGAAn-nTTCn | 4A |
|  | TGTCCAGAAG | 269 | -213 | 1 | 0 | nTTCn-nGAAn | 4A |
|  | ACAATATTCTAAAAA | 99 | -383 | 2 | 0 | nGAAn-nTTCn-nGAAn | 4A |
|  | ATAATATTTCAGAAA | 153 | -329 | 2 | 0 | nGAAn-nTTCn-nGAAn | 4A |
|  | AAACAAGAACCTTCG | 310 | -172 | 2 | 0 | nTTCn-nGAAn-nTTCn | 4A |
|  | TTAACATTTCA | 65 | -417 | 1 | 1 | nGAAn-nTTCn | 4B |
|  | ATAATATTTCA | 153 | -329 | 1 | 1 | nGAAn-nTTCn | 4B |
|  | AAAAGTATTTTTTGAAA | 76 | -406 | 2 | 1 | nGAAn-nTTCn-nGAAn | 4B |
|  | AAAATACTTTCC | 111 | -371 | 1 | 2 | nGAAn-nTTCn | 4B |
|  | TAAAGAATTTCT | 210 | -272 | 1 | 2 | nGAAn-nTTCn | 4B |
|  | AATCATCTGAAA | 359 | -123 | 1 | 2 | nTTCn-nGAAn | 4B |

**Supplementary Table S5c.** HSE motifs call using Python code (<https://github.com/qiandemoni/HSE_sequence_finder>) for *pHvHSP17.7* (a), *pGmHSP17.5* (b), *pZmHSP17.7* (c), and *pZmHSP26* (d).

| **Sequence ID** | **Found Subsequence (mismatch in red)** | **Start Position** | **Transcript Start Site Position** | **Mismatch Count** | **Insertion Size** | **Pattern** | **Ranking** |
| --- | --- | --- | --- | --- | --- | --- | --- |
| pZmHSP17.7 | TTTCCAGAAA | 698 | -1255 | 0 | 0 | nTTCn-nGAAn | 1 |
|  | TATATTTTCCAGAAA | 693 | -1260 | 2 | 0 | nGAAn-nTTCn-nGAAn | 1 |
|  | AGAACTCTCCAGAAA | 1763 | -190 | 1 | 0 | nGAAn-nTTCn-nGAAn | 2A |
|  | GGAACCTGCA | 185 | -1768 | 1 | 0 | nGAAn-nTTCn | 2A |
|  | TGATGGTTCT | 239 | -1714 | 1 | 0 | nGAAn-nTTCn | 2A |
|  | TGAACAGTCA | 795 | -1158 | 1 | 0 | nGAAn-nTTCn | 2A |
|  | AGGAACTTCC | 1114 | -839 | 1 | 0 | nGAAn-nTTCn | 2A |
|  | GGAACTTCCG | 1115 | -838 | 1 | 0 | nGAAn-nTTCn | 2A |
|  | AGAACTCTCC | 1763 | -190 | 1 | 0 | nGAAn-nTTCn | 2A |
|  | AGAAACTGCT | 1773 | -180 | 1 | 0 | nGAAn-nTTCn | 2A |
|  | GTTCACAAAC | 13 | -1940 | 1 | 0 | nTTCn-nGAAn | 2A |
|  | TTTCGCGAGA | 268 | -1685 | 1 | 0 | nTTCn-nGAAn | 2A |
|  | GTTGGTGAAG | 291 | -1662 | 1 | 0 | nTTCn-nGAAn | 2A |
|  | TTTCAGGGAG | 379 | -1574 | 1 | 0 | nTTCn-nGAAn | 2A |
|  | ATTTCTGAAC | 790 | -1163 | 1 | 0 | nTTCn-nGAAn | 2A |
|  | GTTCAGGAGC | 926 | -1027 | 1 | 0 | nTTCn-nGAAn | 2A |
|  | TTTCCAGCAT | 1600 | -353 | 1 | 0 | nTTCn-nGAAn | 2A |
|  | GTCCATGAAC | 1624 | -329 | 1 | 0 | nTTCn-nGAAn | 2A |
|  | TTACTAGAAA | 1638 | -315 | 1 | 0 | nTTCn-nGAAn | 2A |
|  | ATCCGCGAAC | 1748 | -205 | 1 | 0 | nTTCn-nGAAn | 2A |
|  | CTTCTAGGAA | 1829 | -124 | 1 | 0 | nTTCn-nGAAn | 2A |
|  | CTTCCCCAAA | 1898 | -55 | 1 | 0 | nTTCn-nGAAn | 2A |
|  | TTTCCAGAAATATGG | 698 | -1255 | 2 | 0 | nTTCn-nGAAn-nTTCn | 2A |
|  | ATTTCTGAACAGTCA | 790 | -1163 | 2 | 0 | nTTCn-nGAAn-nTTCn | 2A |
|  | TTCCCCAAATCTTCG | 1899 | -54 | 2 | 0 | nTTCn-nGAAn-nTTCn | 2A |
|  | CTTCTAGGAAT | 1829 | -124 | 0 | 1 | nTTCn-nGAAn | 2B |
|  | AGACCCCTTCTAGGAAT | 1823 | -130 | 1 | 1 | nGAAn-nTTCn-nGAAn | 3 |
|  | CGAACCACTCT | 67 | -1886 | 1 | 1 | nGAAn-nTTCn | 3 |
|  | CGAAGAGTTGT | 134 | -1819 | 1 | 1 | nGAAn-nTTCn | 3 |
|  | CGAATCTATCC | 357 | -1596 | 1 | 1 | nGAAn-nTTCn | 3 |
|  | TGCAGAGTTCA | 920 | -1033 | 1 | 1 | nGAAn-nTTCn | 3 |
|  | GGAAAGGATCT | 961 | -992 | 1 | 1 | nGAAn-nTTCn | 3 |
|  | GGAATTTGTCC | 1434 | -519 | 1 | 1 | nGAAn-nTTCn | 3 |
|  | TGAATTATTTT | 1591 | -362 | 1 | 1 | nGAAn-nTTCn | 3 |
|  | AGAACTCTCCA | 1763 | -190 | 1 | 1 | nGAAn-nTTCn | 3 |
|  | AGACCCCTTCT | 1823 | -130 | 1 | 1 | nGAAn-nTTCn | 3 |
|  | TTTCAGGGAGA | 379 | -1574 | 1 | 1 | nTTCn-nGAAn | 3 |
|  | TTTAACAGAAA | 559 | -1394 | 1 | 1 | nTTCn-nGAAn | 3 |
|  | TTTCGCACAAT | 655 | -1298 | 1 | 1 | nTTCn-nGAAn | 3 |
|  | TTTTCCAGAAA | 697 | -1256 | 1 | 1 | nTTCn-nGAAn | 3 |
|  | TTTCCAGAAAT | 698 | -1255 | 1 | 1 | nTTCn-nGAAn | 3 |
|  | GTACAGTGAAA | 769 | -1184 | 1 | 1 | nTTCn-nGAAn | 3 |
|  | ATTAACCGAAA | 806 | -1147 | 1 | 1 | nTTCn-nGAAn | 3 |
|  | ATTACTAGAAA | 1637 | -316 | 1 | 1 | nTTCn-nGAAn | 3 |
|  | ATTCACTCAAA | 1884 | -69 | 1 | 1 | nTTCn-nGAAn | 3 |
|  | CTTCCCCAAAT | 1898 | -55 | 1 | 1 | nTTCn-nGAAn | 3 |
|  | CTTCGTTGACA | 1921 | -32 | 1 | 1 | nTTCn-nGAAn | 3 |
|  | CTTCGATTAGATCTTCG | 1909 | -44 | 2 | 1 | nTTCn-nGAAn-nTTCn | 3 |
|  | ATTCACTCAAACCTCTTCC | 1884 | -69 | 1 | 2 | nTTCn-nGAAn-nTTCn | 3 |
|  | AGAACCAAATCC | 277 | -1676 | 1 | 2 | nGAAn-nTTCn | 3 |
|  | CGAATCTATCCG | 357 | -1596 | 1 | 2 | nGAAn-nTTCn | 3 |
|  | GGAACAAATTAG | 511 | -1442 | 1 | 2 | nGAAn-nTTCn | 3 |
|  | GGAACAGAATCA | 712 | -1241 | 1 | 2 | nGAAn-nTTCn | 3 |
|  | AGCATTATTTCT | 784 | -1169 | 1 | 2 | nGAAn-nTTCn | 3 |
|  | AGAATTGCTTGA | 863 | -1090 | 1 | 2 | nGAAn-nTTCn | 3 |
|  | CGAGGAACTTCC | 1112 | -841 | 1 | 2 | nGAAn-nTTCn | 3 |
|  | GGAATTTGTCCT | 1434 | -519 | 1 | 2 | nGAAn-nTTCn | 3 |
|  | TGAATTATTTTT | 1591 | -362 | 1 | 2 | nGAAn-nTTCn | 3 |
|  | TTTCGCGAGAGA | 268 | -1685 | 1 | 2 | nTTCn-nGAAn | 3 |
|  | TTTAGGGTGAAG | 465 | -1488 | 1 | 2 | nTTCn-nGAAn | 3 |
|  | TTTTAACAGAAA | 558 | -1395 | 1 | 2 | nTTCn-nGAAn | 3 |
|  | TTTCAGCAGCAC | 583 | -1370 | 1 | 2 | nTTCn-nGAAn | 3 |
|  | ATTTTCCAGAAA | 696 | -1257 | 1 | 2 | nTTCn-nGAAn | 3 |
|  | GTTCAGGAGCAA | 926 | -1027 | 1 | 2 | nTTCn-nGAAn | 3 |
|  | CTTCCCACGACT | 1479 | -474 | 1 | 2 | nTTCn-nGAAn | 3 |
|  | CTTAATCCGAAG | 1520 | -433 | 1 | 2 | nTTCn-nGAAn | 3 |

**Supplementary Table S5c.** HSE motifs call using Python code (<https://github.com/qiandemoni/HSE_sequence_finder>) for *pHvHSP17.7* (a), *pGmHSP17.5* (b), *pZmHSP17.7* (c), and *pZmHSP26* (d). (Continued)

| **Sequence ID** | **Found Subsequence (mismatch in red)** | **Start Position** | **Transcript Start Site Position** | **Mismatch Count** | **Insertion Size** | **Pattern** | **Ranking** |
| --- | --- | --- | --- | --- | --- | --- | --- |
| pZmHSP17.7 | GTTGAGCTGAAT | 1584 | -369 | 1 | 2 | nTTCn-nGAAn | 3 |
|  | ATTCACTCAAAC | 1884 | -69 | 1 | 2 | nTTCn-nGAAn | 3 |
|  | GGAATTTGTCCTCACGACT | 1434 | -519 | 2 | 2 | nGAAn-nTTCn-nGAAn | 3 |
|  | TGAATTATTTTTCCAGCAT | 1591 | -362 | 2 | 2 | nGAAn-nTTCn-nGAAn | 3 |
|  | TGAACAAAATTACTAGAAA | 1629 | -324 | 2 | 2 | nGAAn-nTTCn-nGAAn | 3 |
|  | GTTGAGCTGAATTATTTTT | 1584 | -369 | 2 | 2 | nTTCn-nGAAn-nTTCn | 3 |
|  | CAAATCTTCG | 1904 | -49 | 1 | 0 | nGAAn-nTTCn | 4A |
|  | TCTCCAGAAA | 1768 | -185 | 1 | 0 | nTTCn-nGAAn | 4A |
|  | CAAAATTACTAGAAA | 1633 | -320 | 2 | 0 | nGAAn-nTTCn-nGAAn | 4A |
|  | TCTCCAGAAACTGCT | 1768 | -185 | 2 | 0 | nTTCn-nGAAn-nTTCn | 4A |
|  | CCAAATCTTCG | 1903 | -50 | 1 | 1 | nGAAn-nTTCn | 4B |
|  | AATCTGAGAAT | 213 | -1740 | 1 | 1 | nTTCn-nGAAn | 4B |
|  | GGTCCATGAAC | 1623 | -330 | 1 | 1 | nTTCn-nGAAn | 4B |
|  | TATCCGCGAAC | 1747 | -206 | 1 | 1 | nTTCn-nGAAn | 4B |
|  | TCAATCAATTCA | 1877 | -76 | 1 | 2 | nGAAn-nTTCn | 4B |
|  | CAAACCTCTTCC | 1891 | -62 | 1 | 2 | nGAAn-nTTCn | 4B |
|  | GGTCTGAGGAAT | 1558 | -395 | 1 | 2 | nTTCn-nGAAn | 4B |
|  | ACTCTCCAGAAA | 1766 | -187 | 1 | 2 | nTTCn-nGAAn | 4B |
|  | ATAATGATTTTAACAGAAA | 551 | -1402 | 2 | 2 | nGAAn-nTTCn-nGAAn | 4B |
|  | AAAATATATTTTCCAGAAA | 689 | -1264 | 2 | 2 | nGAAn-nTTCn-nGAAn | 4B |
|  | TCAATCAATTCACTCAAAC | 1877 | -76 | 2 | 2 | nGAAn-nTTCn-nGAAn | 4B |

**Supplementary Table S5d.** HSE motifs call using Python code (<https://github.com/qiandemoni/HSE_sequence_finder>) for *pHvHSP17.7* (a), *pGmHSP17.5* (b), *pZmHSP17.7* (c), and *pZmHSP26* (d).

| **Sequence ID** | **Found Subsequence (mismatch in red)** | **Start Position** | **Transcript Start Site Position** | **Mismatch Count** | **Insertion Size** | **Pattern** | **Ranking** |
| --- | --- | --- | --- | --- | --- | --- | --- |
| pZmHSP26 | AGAATGTTCA | 535 | -304 | 0 | 0 | nGAAn-nTTCn | 1 |
|  | AGAATGTTCAAGAGA | 535 | -304 | 1 | 0 | nGAAn-nTTCn-nGAAn | 1 |
|  | GCACGAGAATGTTCA | 530 | -309 | 2 | 0 | nTTCn-nGAAn-nTTCn | 1 |
|  | GGTAGCTTCA | 634 | -205 | 1 | 0 | nGAAn-nTTCn | 2A |
|  | GTTCAAAAAT | 298 | -541 | 1 | 0 | nTTCn-nGAAn | 2A |
|  | GTTCTAAAAC | 384 | -455 | 1 | 0 | nTTCn-nGAAn | 2A |
|  | GTTCAAGAGA | 540 | -299 | 1 | 0 | nTTCn-nGAAn | 2A |
|  | TTTCCCCATCCTTCC | 649 | -190 | 2 | 0 | nTTCn-nGAAn-nTTCn | 2A |
|  | GGATGGGTTCC | 61 | -778 | 1 | 1 | nGAAn-nTTCn | 3 |
|  | CGAACTGTTTT | 118 | -721 | 1 | 1 | nGAAn-nTTCn | 3 |
|  | TGATAAGTTCA | 449 | -390 | 1 | 1 | nGAAn-nTTCn | 3 |
|  | CGAAGCTCTCT | 585 | -254 | 1 | 1 | nGAAn-nTTCn | 3 |
|  | GTTCCCGGCAA | 67 | -772 | 1 | 1 | nTTCn-nGAAn | 3 |
|  | GTTCCCAGATC | 803 | -36 | 1 | 1 | nTTCn-nGAAn | 3 |
|  | GGATGGGTTCCCGGCAA | 61 | -778 | 2 | 1 | nGAAn-nTTCn-nGAAn | 3 |
|  | AGAATGTTCAAGAGAAG | 535 | -304 | 2 | 1 | nGAAn-nTTCn-nGAAn | 3 |
|  | TGAATAAGTTCA | 291 | -548 | 0 | 2 | nGAAn-nTTCn | 2B |
|  | GTTCAAGAGAAG | 540 | -299 | 0 | 2 | nTTCn-nGAAn | 2B |
|  | CGAGAATGTTCAAGAGAAG | 533 | -306 | 1 | 2 | nGAAn-nTTCn-nGAAn | 3 |
|  | CGGATGGGTTCC | 60 | -779 | 1 | 2 | nGAAn-nTTCn | 3 |
|  | CGAACTGTTTTA | 118 | -721 | 1 | 2 | nGAAn-nTTCn | 3 |
|  | CGAGAATGTTCA | 533 | -306 | 1 | 2 | nGAAn-nTTCn | 3 |
|  | GTTCCCGGCAAG | 67 | -772 | 1 | 2 | nTTCn-nGAAn | 3 |
|  | CTTCCACGGCAC | 659 | -180 | 1 | 2 | nTTCn-nGAAn | 3 |
|  | CTTCGCAAGCAC | 732 | -107 | 1 | 2 | nTTCn-nGAAn | 3 |
|  | CGGATGGGTTCCCGGCAAG | 60 | -779 | 2 | 2 | nGAAn-nTTCn-nGAAn | 3 |
|  | GTATAACTGAATAAGTTCA | 284 | -555 | 2 | 2 | nTTCn-nGAAn-nTTCn | 3 |
|  | CGTCGCGAAG | 580 | -259 | 1 | 0 | nTTCn-nGAAn | 4A |
|  | TGTCCGGTAGCTTCA | 629 | -210 | 2 | 0 | nTTCn-nGAAn-nTTCn | 4A |
|  | CAAAGCGCTTCG | 725 | -114 | 1 | 2 | nGAAn-nTTCn | 4B |
|  | CAAAGCGCTTCGCAAGCAC | 725 | -114 | 2 | 2 | nGAAn-nTTCn-nGAAn | 4B |

**Supplementary Table S6.** GUS activity in second dewlap leaf of the selected HSP lines before any treatment

| **Line ID** | **Before treatment leaf GUS acitivity** | | |
| --- | --- | --- | --- |
|  | **Tip** | **Mid** | **Base** |
| Zm17.7_6 (1) | 0.03±0.01 | 0.03±0.01 | 0.04±0.01 |
| Zm17.7_9 (2) | 0.02±0.00 | 0.02±0.00 | 0.02±0.00 |
| Zm17.7_13(1) | 0.00±0.00 | 0.00±0.00 | 0.00±0.00 |
| Zm17.7_16(4) | 0.02±0.01 | 0.03±0.01 | 0.03±0.00 |
| Gm17.5_3 (1) | 0.01±0.00 | 0.00±0.00 | 0.00±0.00 |
| Gm17.5_5 (1) | 0.00±0.00 | 0.01±0.00 | 0.01±0.00 |
| Gm17.5_6 (2) | 0.01±0.00 | 0.01±0.00 | 0.01±0.00 |
| Gm17.5_13(4) | 0.10±0.01 | 0.00±0.00 | 0.00±0.00 |
| Gm17.5_17(1) | 0.01±0.00 | 0.00±0.00 | 0.01±0.00 |
| Hv17_3 (2) | 0.01±0.00 | 0.01±0.00 | 0.02±0.00 |
| Hv17_4 (2) | 0.02±0.00 | 0.02±0.01 | 0.03±0.01 |
| Hv17_12(1) | 0.01±0.00 | 0.05±0.00 | 0.06±0.01 |
| Hv17_13(4) | 0.04±0.00 | 0.06±0.01 | 0.04±0.02 |
| Hv17_16(1) | 0.08±0.03 | 0.05±0.00 | 0.07±0.02 |
| Zm26_2(2) | 0.01±0.00 | 0.01±0.00 | 0.01±0.00 |
| Zm26_3(1) | 0.01±0.00 | 0.01±0.00 | 0.01±0.00 |
| Zm26_6(2) | 0.01±0.00 | 0.01±0.00 | 0.02±0.01 |
| Zm26_8(2) | 0.00±0.00 | 0.00±0.00 | 0.01±0.01 |
| Zm26_9(1) | 0.08±0.00 | 0.01±0.00 | 0.01±0.00 |
| WT | 0.01±0.00 | 0.01±0.00 | 0.03±0.00 |

Unit: p mol/(min · mg). Data is shown as average ± standard error. (n=3). GUS copy numbers are presented after the line IDs in parentheses.
